# Kawaii emotions in presentations: Viewing a physical touch affects perception of affiliative feelings of others toward an object

**DOI:** 10.1371/journal.pone.0264736

**Published:** 2022-03-24

**Authors:** Yuka Okada, Mitsuhiko Kimoto, Takamasa Iio, Katsunori Shimohara, Hiroshi Nittono, Masahiro Shiomi

**Affiliations:** 1 Department of Agent Interaction Design Laboratory, Advanced Telecommunications Research Institute International, Kyoto, Japan; 2 Department of Information Systems Design, Doshisha University, Kyoto, Japan; 3 Department of Information and Computer Science, Keio University, Kanagawa, Japan; 4 Faculty of Culture and Information Science, Doshisha University, Kyoto, Japan; 5 Graduate School of Human Science, Osaka University, Suita, Japan; The University of Electro-Communications, JAPAN

## Abstract

We investigated how a presenter’s touching behaviors of an object during its explanation affect the observer’s perceived feelings of *kawaii*, a Japanese word that means “cute,” toward the object and the presenter. We conducted a face-to-face experiment with a robot presenter as well as a web survey experiment with both robot and human presenters. Based on the phenomenon that people more firmly touch an object when their perceived *kawaii* feeling is overwhelmingly strong, we investigated the effects of touching behavior with emphasized styles. First, we conducted a face-to-face experiment with a robot presenter where participants observed their presentations about an object to explain its characteristics. The results showed that participants who observed the robot’s touch behaviors perceived the object to be more *kawaii* and thought that the robot also felt the object was more *kawaii*. On the other hand, the results did not effectively show any increase in the participant’s feelings of *kawaii* toward the robot or the emphasized touch style. Based on these results, we next conducted a web survey experiment to investigate whether such knowledge about touching effects is applicable for human presenters. The results resembled those obtained when the presenter was a robot, i.e., viewing a touch behavior increased both the presenter’s perceived feelings of *kawaii* toward the object and the participant’s feelings of *kawaii* toward it. These results suggest that viewing the touch behaviors of others influenced the perceived emotional feelings toward both presenters and objects.

## Introduction

Several studies have reported that people are receptive to cuteness because it promotes positive emotions and behavior changes [1–5]. Cuteness encourages human interaction, such as when a baby’s behavior brings a smile to a parent’s face, when a grandchild’s words change her grandparents’ behaviors, or when strangers share a moment of conversation while they are walking their dogs.

Past studies have also described the importance of the concept of cuteness for robots that operate in daily settings [6, 7]. Such robots typically address the feeling of *kawaii* (a Japanese word that means “cute” [5, 8]) in two ways: 1) enhancing the inherent feeling of *kawaii* in appearances and behaviors, and 2) conveying its feeling to others through behaviors. The first type has already been adopted for consumer objectives, including many recent commercial robots whose appearances are *kawaii* and engage in such behaviors. For example, many companies focus on the positive connotation of *kawaii* when designing the appearance and behavior of robots in Japan. Paro, LOVOT, and Robohon are typical examples. The concept of *kawaii* has become an important element in Japan’s commercial aspects and its pop culture [9, 10]. Related to this approach, the baby scheme [11, 12] is a well-known concept in *kawaii* feelings. Although this scheme has been adopted in existing commercial products, it mainly focuses on the appearance perspective. Thus, unlike the first approach, the behavior designs for conveying a feeling of *kawaii* (i.e., the second approach) have received less research focus. To the best of our knowledge, just one past study investigated possible locomotion behaviors to express *kawaii* feelings using a mobile robot (Roomba) [13].

**Fig 1:**
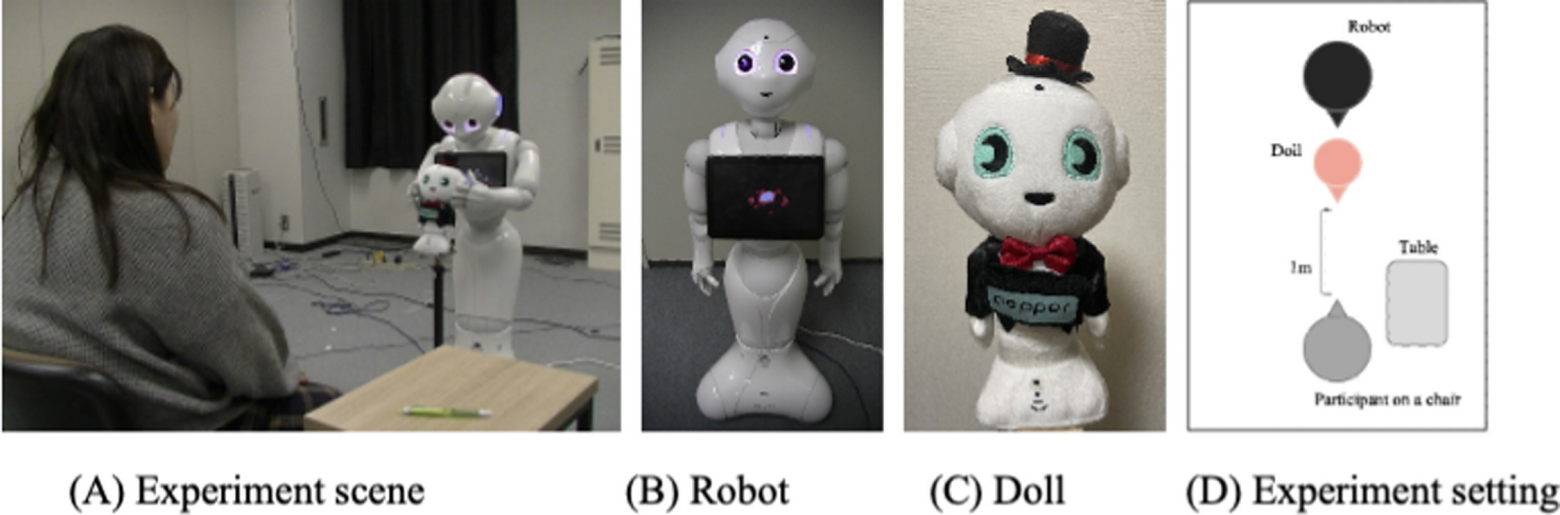
Robot explains a doll with a touching behavior.

Similar to that past study that investigated *kawaii* feelings by locomotion behaviors [13], we are also interested in a robot’s behavior design that conveys the feelings of the *kawaii* qualities of specific things to people (Fig 1A). Such a behavior design might effectively provide information to people, which is a common task for social robots [14–16]. For example, a social robot working as a sales clerk in a shopping mall recommends products by explaining the deliciousness of food, the convenience of gadgets, the economy of things, and the cuteness of dolls. In this context, designing affective social robots becomes essential to achieve natural and smooth interaction with people through their emotional expressions [17–19]. Another study described the relationships between the participants’ attributions of different emotions to robots and their anthropomorphism [20]; affective and emotional behaviors (e.g., expressing *kawaii* feeling by robots) positively affected the perceived anthropomorphism of robots as well as the perceived impressions [21, 22]. Therefore, expressing *kawaii* feelings is effective for robots that interact with people.

**Fig 2:**
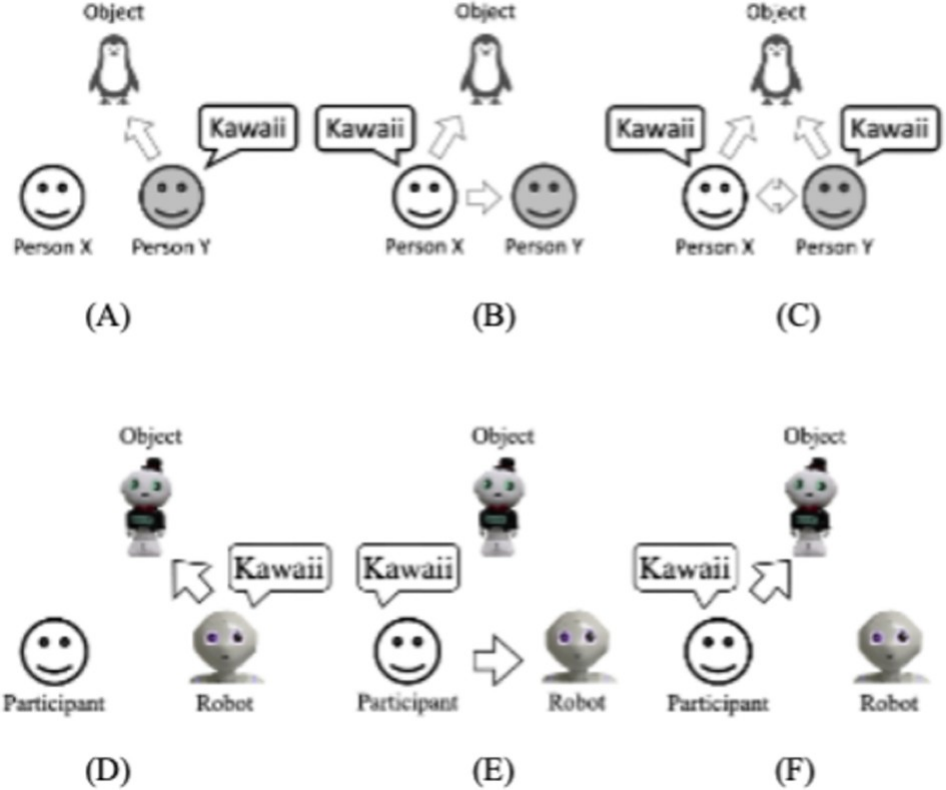
Concept of *kawaii* triangle [8] and our hypotheses based on it.

To emphasize the *kawaii* nature of things through robots, we focused on a concept of social effects called the "*kawaii* triangle" (Fig 2) [8]. If X observes Y’s smile, which was caused by a *kawaii* feeling (Fig 2A) of a certain item (e.g., a penguin), X will probably have a positive impression of that same penguin and the same feeling toward Y (Fig 2B). Expressing a *kawaii* feeling by X will enhance Y’s kawaii feeling, too (Fig 2C). Based on this idea, we assumed that if a social robot expresses more *kawaii* feelings toward a particular item (Fig 2D), then an observer will also have a deeper feeling of *kawaii* toward the social robot and the object (Fig 2E and 2F). Investigating the *kawaii* feelings from a robot presenter to observers is beyond the scope of this study.

To enable the robot to express a feeling of *kawaii* toward an object, we adopted a touching behavior during its explanations. Since people are generally motivated to approach an object due to a feeling of *kawaii* [8], a touching behavior creates a situation where a part of the person’s body (i.e., hand) is close to the object. Based on the *kawaii*-triangle concept, we believe that an observer will feel more of the robot’s *kawaii* feeling toward an object when the robot touches it; the observer will feel deeper *kawaii* feelings toward the object and the robot.

In addition, past studies confirmed “cute aggression,” which describes the relationship between aggressive behaviors and perceived strong *kawaii* feelings [23, 24]. For example, a participant reported that "looking at this baby makes me want to pinch her cheeks and be playfully aggressive" [24]. Therefore, we also assumed that if a social robot touches an object in an exaggerated way, the participants will have a stronger feeling of *kawaii* toward both the robot and the object.

In this study, we examined whether viewing touch behaviors and exaggerated behavior toward an object by a social robot enhanced the robot’s feelings of *kawaii* toward the object and the participant’s feelings of *kawaii* toward both the object and the robot. With a robot named Pepper and a Pepper doll as objects, we used two explanatory behaviors (touch factor: *touch* and *no-touch*) and two action types (motion factor: *normal* and *emphasis*) to address the following research questions in Experiment I.

- Research question 1: Does viewing a robot’s touching behavior enhance the human perception of the robot’s *kawaii* feelings toward the object and the observer’s *kawaii* feelings toward the robot and the object?
- Research question 2: Does viewing an exaggerated explanatory behavior intensify the perception of the robot’s *kawaii* feelings toward an object? Does it induce more *kawaii* feelings toward the object or the robot in the observer?

We also examined by a web survey whether these pieces of knowledge from Experiment I are applicable for both robot and human presenters who more commonly work in real environments to address the following additional research question in Experiment II:

- Research question 3: Does viewing a human presenter’s touching behavior enhance her *kawaii* feelings toward the object and the observer’s *kawaii* feelings toward both the presenter and her object?.

## Material and method of Experiment I

In this experiment, we investigated how viewing a robot’s touching behaviors and a *cute aggression* design influences the human perception of the robot’s *kawaii* feelings toward the object and the observer’s *kawaii* feelings toward the robot and the object. All procedures were approved by the Advanced Telecommunication Research Review Boards (20-501-4).

### Robot, task, and environment

**Fig 3:**
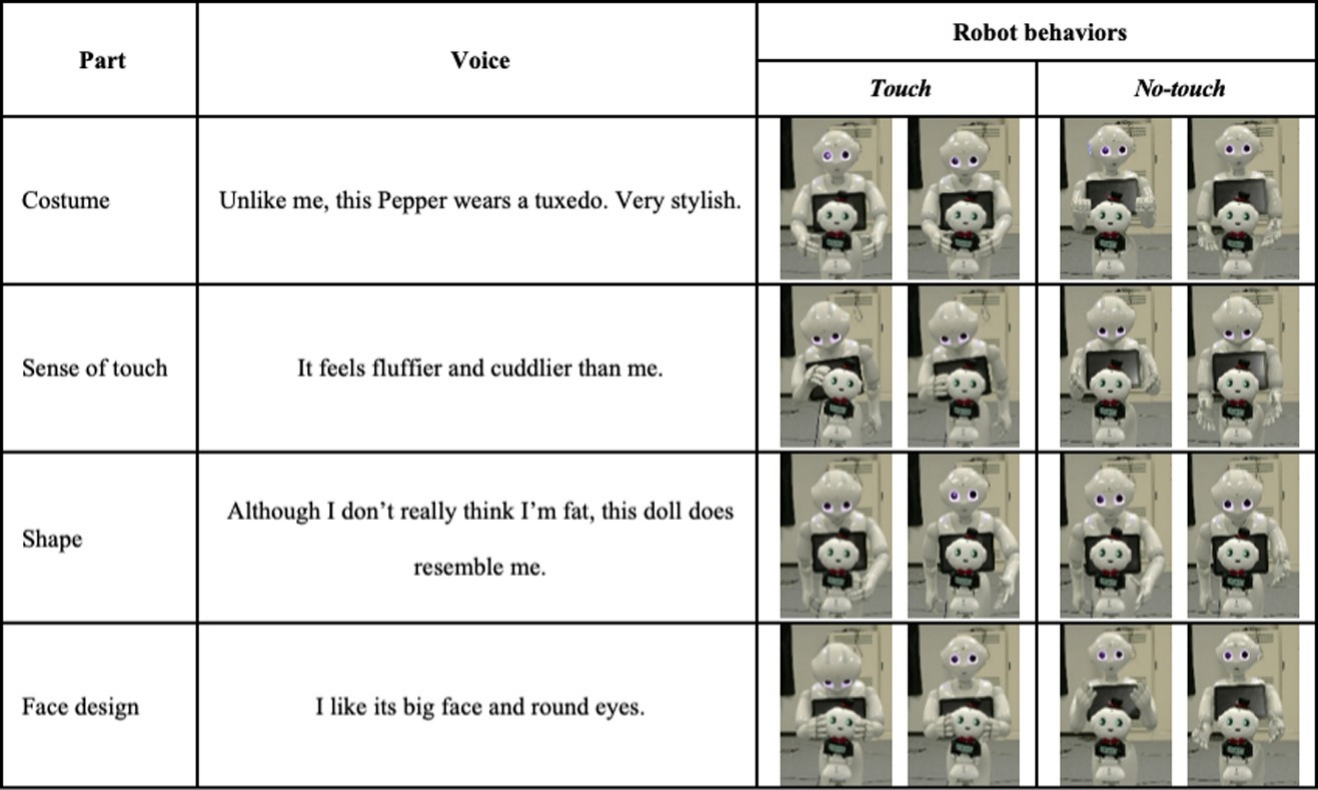
Speech contents and robot behaviors in each condition.

We employed Pepper (Fig 1B) to explain objects in this study. It has 20 degrees of freedom (DOFs): 2 DOFs in its head, 6 in both arms, and 6 in its lower body. It stands 121 cm high. As an object, we also employed a 28-cm-tall Pepper doll (Fig 1C) and placed it between Pepper and the subject (Fig 1D). We adjusted the doll’s height by placing it on a 65-cm high stand. In the informative task, Pepper introduced itself to the participant and explained the four features of the doll: costume, tactile sensation, shape, and facial design. The speech content of each part is shown in Fig 3.

### Conditions

We employed a within-subjects experiment design. The participants in this study joined four trials: two touch factors (*touch* and *no-touch*) and two motion factors (*normal* and *emphasis*). The orders of the experimental conditions were counterbalanced to avoid order effects. Note that in a within-subjects design, carryover effects are possible from one condition to the next. In this case, we used a within-subjects design because we considered the robot’s novelty for the general public; even though we used Pepper, the participants came from different backgrounds and had different impressions of it. Since ceiling and floor effects due to the novelty of robots for ordinary people and the large variance of their perceived impressions between conditions must be avoided in such situations, we chose a within-subjects design to study the effects of both factors.

#### Touch factor

For the *touch* condition, we illustrated the four parts of the doll described above by preparing four touch actions (Fig 3): touching the doll’s body (costume), stroking its head (sense of touch), touching its feet (shape), and squeezing its cheeks (face design).

We prepared four no-touch actions for the no-touch condition (Fig 3) by spreading its hands around the doll’s body, head, and feet. Since past studies investigated the effectiveness of deictic and iconic gestures for information-providing tasks [25–28], we designed the hand motions to describe the shape and attract attention toward the explained parts.

#### Motion factor

For the *normal* condition, we determined the robot’s motion speeds by observing human behaviors. We conducted a preliminary data collection where participants freely explained their perceived *kawaii* feeling of the doll for one minute at our laboratory. Three participants joined the data collection and they showed several touch behaviors during their explanations, such as contacting, stroking, squeezing, etc. Because of the difference between the physical characteristics of people and robots, we heuristically adjusted the motion speeds of the robots’ behaviors.

For the *emphasized* condition, we increased the motion speeds of the robot to emphasize its explanations. We heuristically adjusted the ratios of the motion speeds and chose a speed that is three times faster than the normal condition after several discussions and confirmations among the authors. We focused on representing the gestures, i.e., not on other features related to the speech, although previous studies emphasized the effectiveness of pitch enhancement for information-providing tasks [27, 28]. Considering the concept of cute aggression, this study investigated the effect of the robot’s touch behavior and its style in expressing feelings of *kawaii*, not the effect of integrating gestures and speech.

### Measurement

We used two questionnaire items to investigate the feelings of *kawaii*: the degree of *kawaii* and *wanting to approach* [5, 8]. We used these items because the related studies [5, 8] claimed that the feelings of *kawaii* are related to motivation about wanting to approach. We measured these items for three targets: the robot’s feelings toward the doll, the participant’s feelings toward the doll, and the participant’s feelings toward the robot. We also measured two questionnaire items to investigate the impressions toward the robot’s explanations: the degree of a *good presentation* and the *naturalness of entire motions*. All the items were assessed using a one-to-seven response format, where 1 was the most negative and 7 was the most positive. A free-response form was also provided.

### Procedure

After the participants provided written, informed consent, the researcher clearly explained the experiment’s procedure and asked them to imagine a situation in which a shopkeeper wanted to recommend a doll. The robot’s actions are basically the same in all the conditions except for the touch behaviors and their motion styles. That is, the robot extended a greeting, described the doll, and ended the dialogue in one session. After each session, the participants answered a questionnaire.

### Participants

Forty-two participants, equally divided by gender, ranging in age from 21 to 49, joined our experiment with a mean age of 37.83 years and an S.D. of 7.92.

## Results and discussion of Experiment I

### Questionnaire results

**Fig 4:**
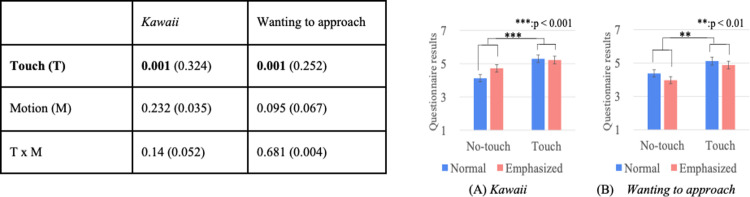
Perceived robot’s feeling of *kawaii* and wanting to approach doll.

**Fig 5:**
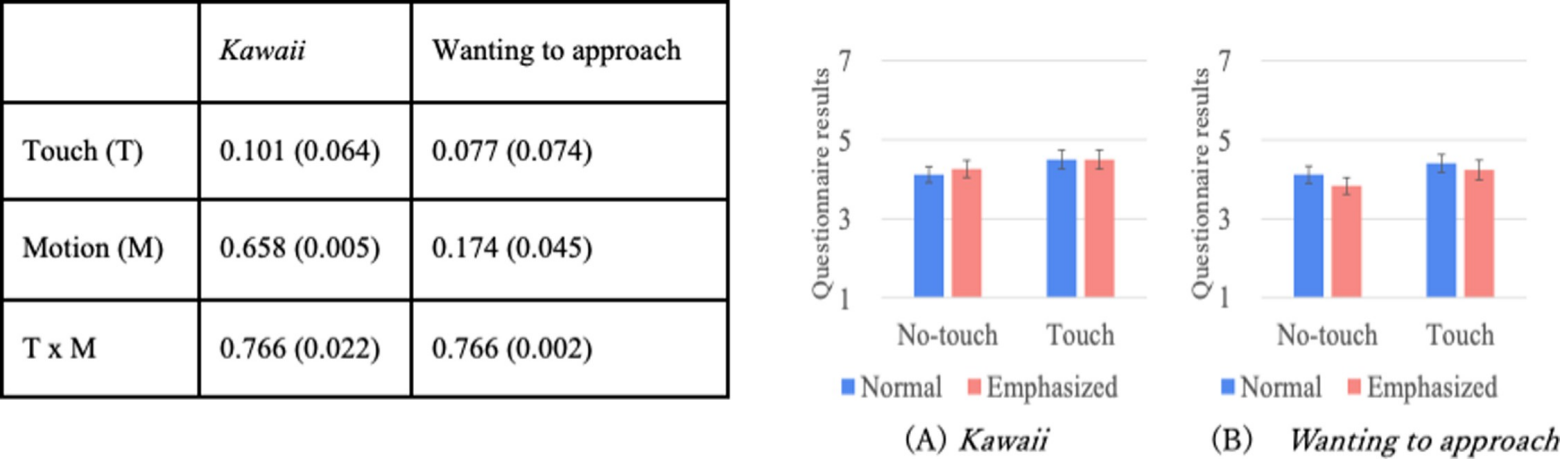
*Kawaii* and wanting to approach robot.

**Fig 6:**
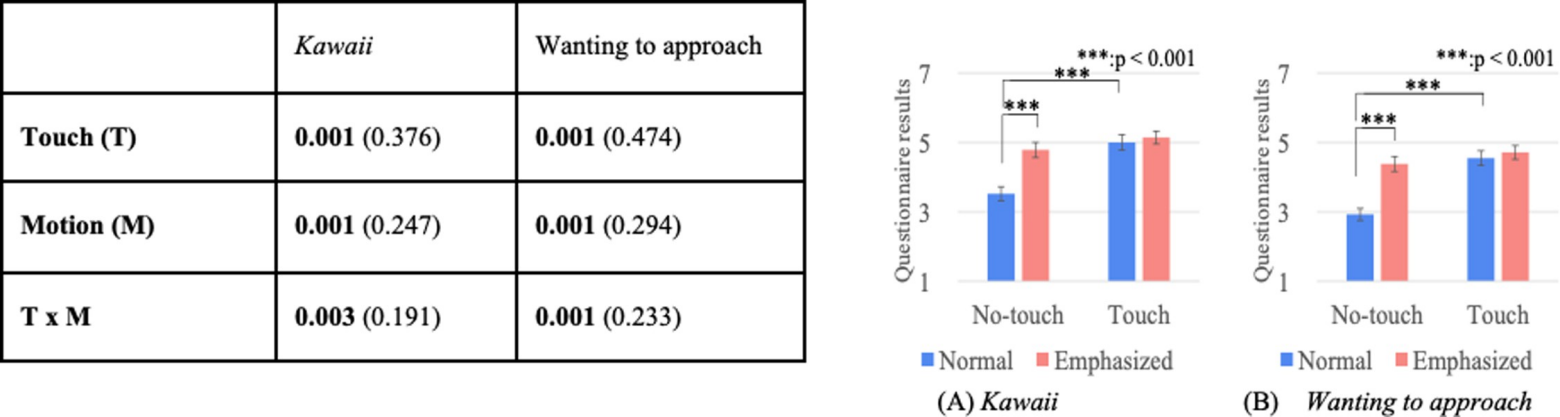
*Kawaii* and wanting to approach doll.

**Fig 7:**
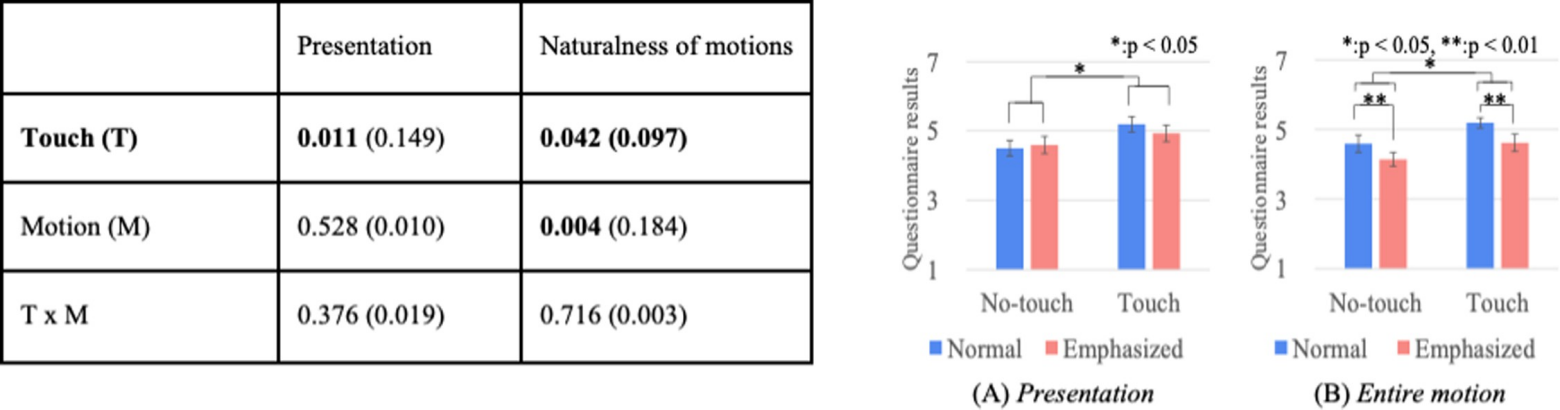
Descriptions of robot’s presentation and naturalness toward motions.

Figs 4–7 show the results of a two-way repeated ANOVA and the graphs (average and S.E.) of the perceived feelings of *kawaii* and *wanting to approach*, robot’s feeling toward the doll, participant’s feeling toward the robot, and participant’s feeling toward the doll. The bold texts of the ANOVA results denote significant differences. Due to a large number of combinations, the texts below only explain the significantly different parts in the analysis.

The analysis results of the perceived robot’s feelings of *kawaii* and *wanting to approach* the doll only showed significant differences in the touch factor (Fig 4). The analysis results of the perceived participant’s feelings of *kawaii* and *wanting to approach* the robot did not show significant differences in all the factors (Fig 5). The analysis results of the perceived participant’s feelings of *kawaii* and *wanting to approach* the doll showed significant differences in all the factors (Fig 6). For *kawaii* feelings, the simple main effects showed significant differences: *touch* > *no-touch*, *p <* 0.001 in the *normal* condition, and *emphasized* > *normal*, *p* < 0.001 in the *no-touch* condition. For *wanting to approach*, the simple main effects showed significant differences: *touch* > *no-touch*, *p <* 0.001 in the *normal* condition, and *emphasized* > *normal*, *p* < 0.001 in the *no-touch* condition. The analysis results of the participants’ feelings about a *good description* and the *naturalness of all the motions* showed significant differences in the touch factor (Fig 7).

### Summary of questionnaire analysis

**Fig 8:**
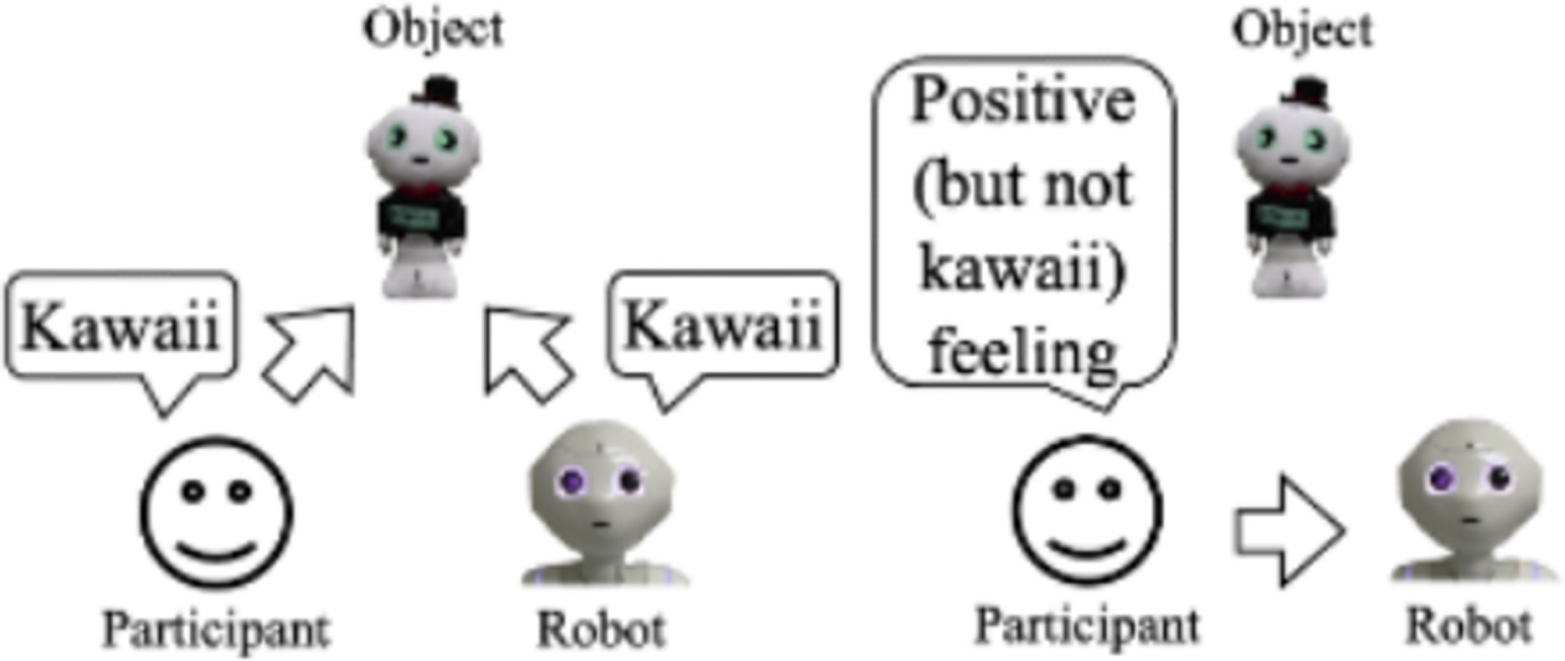
Summary of Experiment I’s results.

We found that the robot’s touching behavior increased the feelings of *kawaii* and wanting to approach the doll more than the non-touching behavior. The robot’s impression of the doll also increased more than the normal non-touching style. In addition, the robot’s explanation was evaluated more highly when the touch behavior was used. Note that the robot’s touch behavior did not increase the participants’ feeling of *kawaii* or wanting to approach the robot compared to the no-touch behavior of the robot in the normal style. Thus, the experiment results showed partial support for research question 1; the touching behavior enhanced the robot’s and the observers’ *kawaii* feelings toward the object, but not the observer’s *kawaii* feelings toward the robot (Fig 8).

In addition, we identified no significant effect of emphasis style on the touch behavior. Thus, the experiment results did not support research question 2; the exaggerated explanatory behaviors did not more deeply intensify the robot’s *kawaii* feelings toward the object. Instead, the emphasis style was effective for the no-touch condition. Compared to the normal style, the no-touch behavior of the robot in the emphasized style increased the *kawaii* feeling and motivation to approach the doll and the participants’ feelings and motivation to approach the doll.

### Additional analysis about free descriptions

We conducted an additional analysis of the free-description results of the questionnaires. Two coders categorized all 167 sentences into three types (positive, negative, or other) for the speech and motion categories (because we prepared different motions among the conditions, this separation is needed to individually analyze their opinions) shown in Table 1. Typical examples of positive categories included natural, polite, and *kawaii*, and typical examples of negative categories included unnatural, exaggerated, and difficult to understand. If a description had two or more different opinions in one category, e.g., “the robot’s explanations made the doll more *kawaii*, but its speeches were unnatural,” we categorized it as “negative,” based on its overall nuance. The kappa coefficient was 0.71, indicating substantial agreement.

**Table 1:** Coding of free-description results.

		Speech			Motion		
		Positive	Negative	Other	Positive	Negative	Other
Normal	No-touch	10	11	3	5	4	0
	Touch	10	8	6	13	13	0
Emphasized	No-touch	14	9	0	3	8	1
	Touch	14	6	2	10	16	1

We conducted a chi-square test for each category with positive/negative items whose results did not show significant differences. However, in the free-description form, note the following comments: “I felt that Pepper thinks the doll is *kawaii*” or “While I was watching the robot’s touch behavior, I also wanted to touch the doll” in the touch conditions. Perhaps these opinions are indirect evidence about how the robot’s touch behaviors increased the participants’ feeling of *kawaii* and their motivation to approach the doll.

## Implication and future works based on Experiment I

Our experimental results suggest several implementation methods for social robots in the context of expressing *kawaii* feelings. The first is the effectiveness of touch behaviors toward objects when a robot explains or recommends them. The participants positively reacted to the explanations with the robot’s touch behaviors. Note that the emphasis style is ineffective for the touch behavior design, although in this study, our robot had several hardware limitations, including a torque limit. Therefore, we haven’t yet deeply investigated the effects of the emphasis style. This topic is one possible future work.

Based on the experiment results, we are interested in whether touching behavior is useful for human presenters to express *kawaii* feelings. As described in the introduction, the main aim of this study is to investigate the effectiveness of touching behavior to express *kawaii* feelings using social robots that provide information to people. If touch behaviors effectively improve such feelings toward an object, such knowledge would be useful for human shopkeepers’ information-providing tasks in the context of expressing *kawaii* feelings. To design an experiment with human presenters, we eliminated the exaggerated explanatory behavior in the second experiment due to the non-significant effects in the touching behaviors and a demerit in the non-touching behaviors (significantly low naturalness). Showing natural and acceptable explanation behaviors is an important requirement in such a context. Based on this consideration, we conducted the following additional experiment.

## Material and method of Experiment II

In Experiment II, we investigated the effectiveness of the human presenter’s touching behaviors toward feelings of *kawaii* and compared them with the robot’s presenter. Based on the results of Experiment I, i.e., where the emphasis style is ineffective for the touch behavior design and both the positive/negative effects for the no-touch behavior design (better for perceived feeling but less naturalness), we did not investigate the cute aggression effect in this experiment. All procedures were approved by the Advanced Telecommunication Research Review Boards (21-501-4).

### Visual stimulus

We took video with the same robot from Experiment I and replaced the actions performed by the robot in the previous experiment with a human presenter. Since the robot lacked the ability to express facial expressions, the human wore a mask to hide her facial expressions. We used the same doll from Experiment I and adjusted the height and position relationship between it and the presenter to be identical. The dialogues were also identical to Experiment I, although we made some slight changes to humanize the explainer. For example, the robot presenter explained that the doll’s design is based on itself, but the human presenter explained that its design is based on a robot named Pepper. Both presenters gave a greeting, explained the doll, and ended the dialogue in each video similar to Experiment I’s robot behaviors. The videos lasted about 30 seconds. As mentioned above, since we did not investigate the cute aggression effect, we prepared two types of behaviors: touch and no-touch. The details of the conditions and behaviors are described in the next section.

### Conditions

We employed a mixed between (presenter factor, *human* or *robot*) and within-subject design (touch factor, *touch*, and *no-touch*). Therefore, half of our participants watched two videos with two touch factors (*touch* and *no-touch*) with a human presenter, and the others watched two videos with two touch factors (*touch* and *no-touch*) with a robot presenter. The order was counterbalanced. We employed a between-subject design for the presenter factor to avoid longer watching times in the web survey, which might cause inappropriate answers.

#### Presenter factor

In the *human* condition, the human presenter described the doll. For the *robot* condition, the robot from Experiment I described it.

#### Touch factor

**Fig 9:**
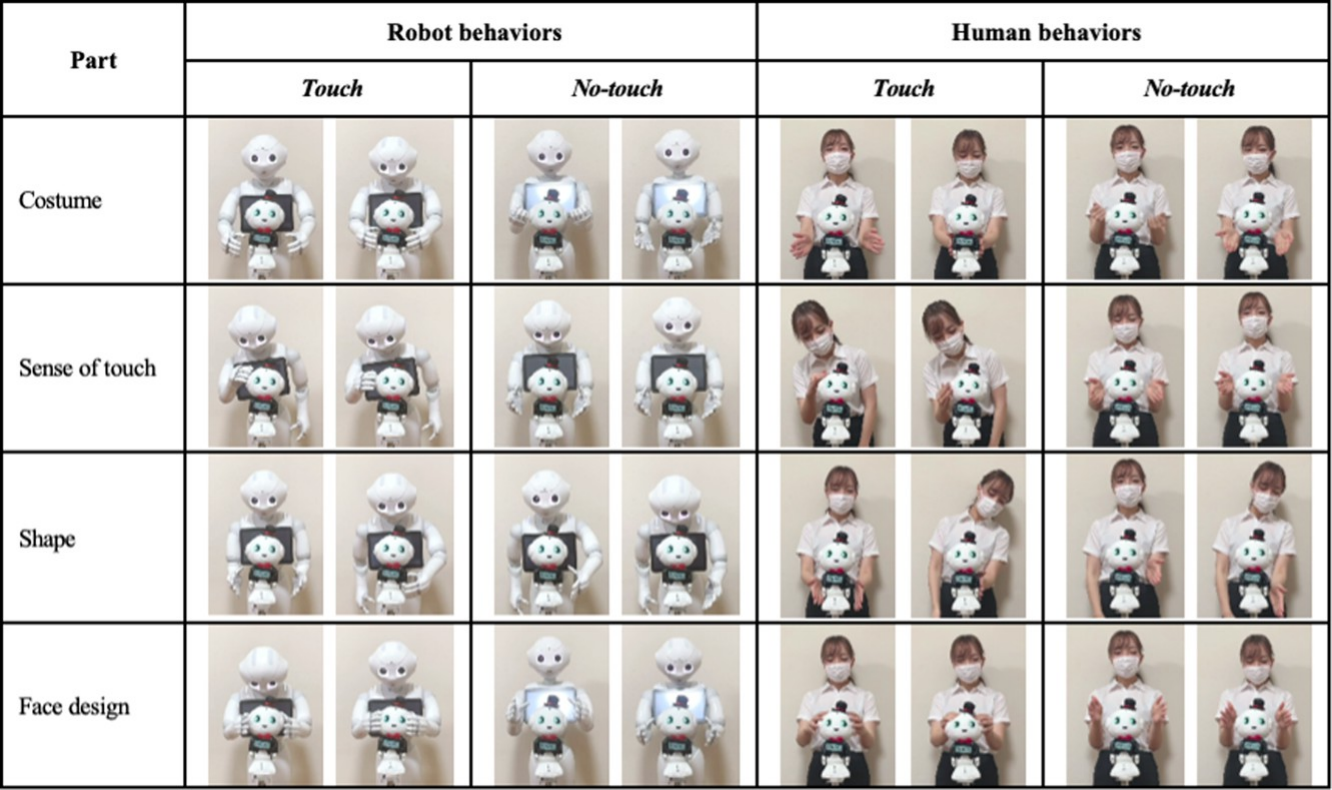
Presenter’s behaviors in each condition.

For the *touch* condition, we prepared four types of touch behaviors based on Experiment I (Fig 9, left). Similar to the robot presenter, the human presenter touched the doll during the explanations. In the *no-touch* condition, we also prepared four types of no-touch behaviors, as in Experiment I (Fig 9, right).

### Procedure

Our survey was conducted online through a web site, and a survey company recruited the participants. First, they read the explanations of the data collection and how to evaluate each video. In this data experiment, they observed videos with different behaviors of the human or robot presenters. We prepared two videos and dummy questions to check whether the participants carefully watched them and the quality of their answers because past researched reported the need for screening participants in a web survey [29, 30].

### Measurement

Similar to Experiment I, we used two questionnaire items to investigate the *kawaii* feelings: the degree of *kawaii* and *wanting to approach* [5, 8]. We measured these items for three targets: the presenter’s feelings toward the doll, the participant’s feelings toward the doll, and the participant’s feelings toward the presenter. All the items were assessed using a one-to-seven response format, where 1 was the most negative and 7 was the most positive. We did not investigate the impressions toward the robot’s explanations in Experiment II to avoid longer answer times.

### Participants

Four hundred and nine participants joined our experiment: 213 females and 189 males (seven declined to specify gender) whose ages ranged from 19 to 74, their average age was 39.65, with an S.D. of 10.18. 207 participants watched the videos with the human presenter, and 202 watched with the robot presenter. After the screening process, 124 valid participants (58 females and 66 males) watched the videos with the human presenter, and 135 (75 females, 58 males, and 2 no answers) watched with the robot presenter.

## Results and discussion of Experiment II

### Questionnaire results

**Fig 10:**
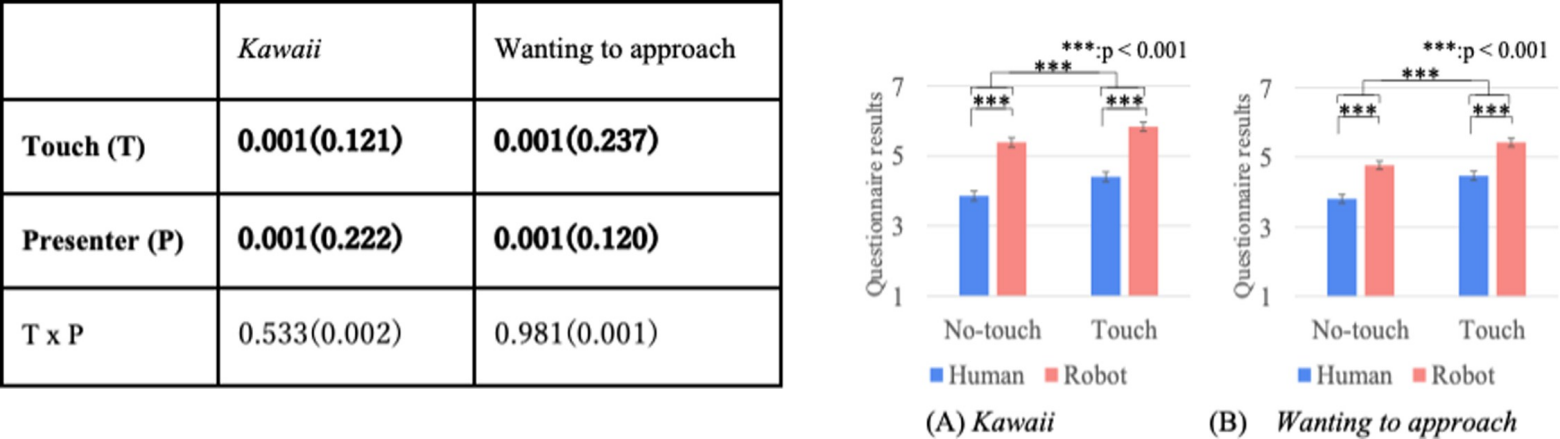
Perceived presenter’s feelings of kawaii and wanting to approach doll.

**Fig 11:**
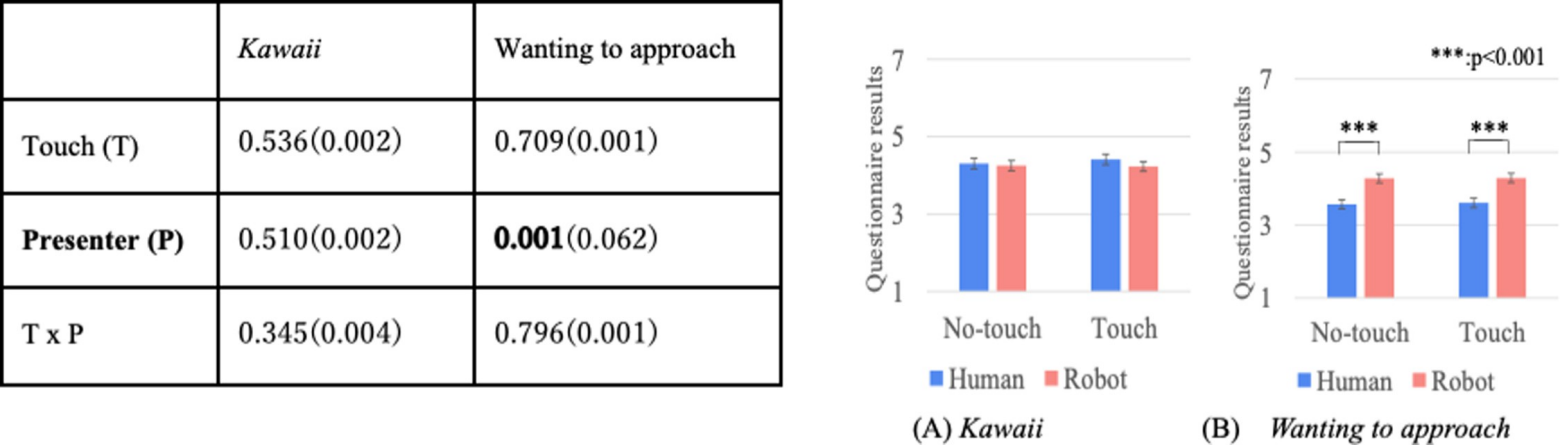
Participants’ feelings of *kawaii* and wanting to approach presenter.

**Fig 12:**
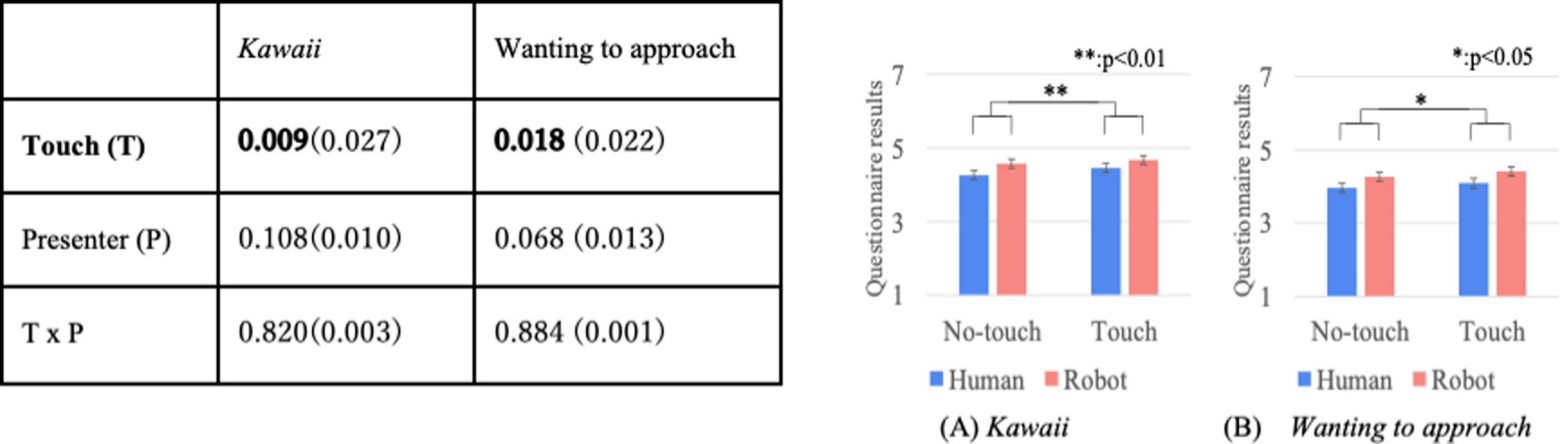
Participants’ feelings of *kawaii* and wanting to approach doll.

Figs 10–12 show the results of a two-way mixed and repeated ANOVA and the graphs (average and S.E.) of the perceived feelings of *kawaii* and *wanting to approach*, the presenter’s feeling toward the doll (Fig 10), the participant’s feeling toward the presenters (Fig 11), and the participant’s feeling toward the doll (Fig 12). The bold texts in table denote significant differences.

The perceived presenter’s feelings of *kawaii* and wanting to approach the doll showed significant differences in the touch (*touch* > *no-touch*, *p<*0.001) and presenter factors (*robot* > *human*, *p*<0.001). The analysis results of the perceived participant’s feelings of *kawaii* and *wanting to approach* the presenter showed a significant difference in the presenter factor (only for *wanting to approach*, *robot* > *human*, *p<*0.001). The analysis results of the perceived participant’s feelings of *kawaii* and *wanting to approach* the doll showed significant differences in the touch factor (*touch* > *no-touch*, *p*<0.01). Note that the analysis results did not show any significant differences in the interaction effects between the touch and presenter factors for all questionnaire items.

### Summary of questionnaire analysis

**Fig 13:**
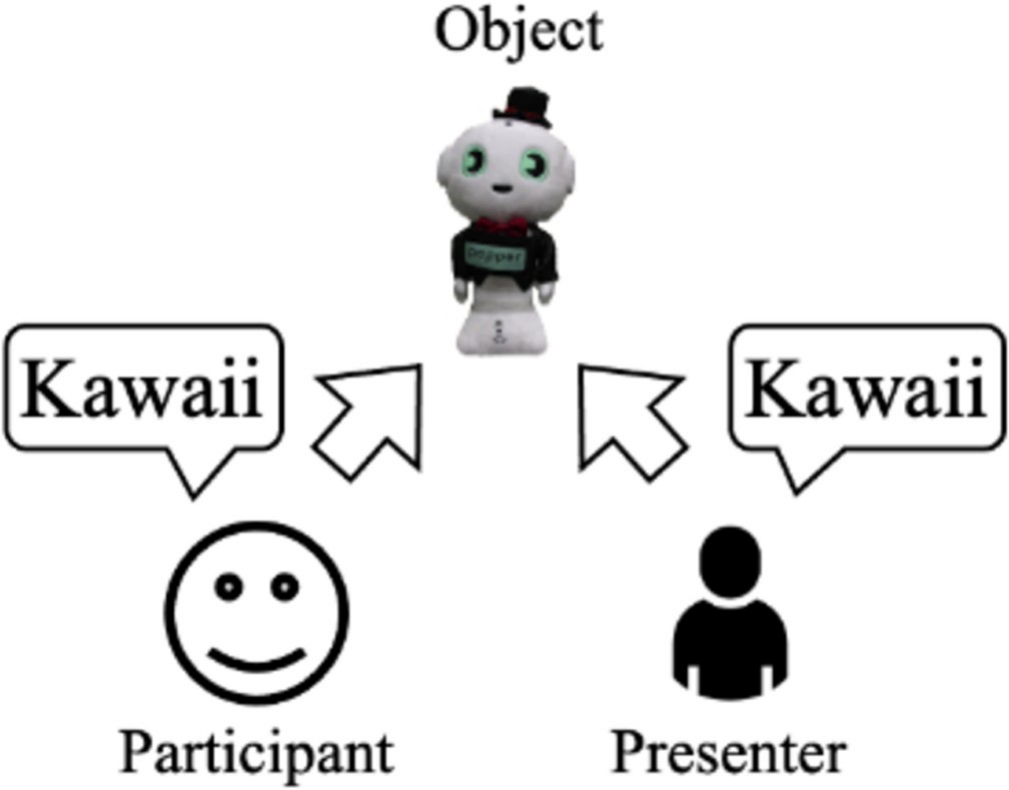
Summary of Experiment II’s results.

We found that the presenter’s touching behavior increased the feelings of *kawaii* and wanting to approach the doll compared to the non-touching behavior for both the human/robot presenters. Participants evaluated the human presenter relatively lower than the robot presenter. The trends of the touch factor effects are similar between the human and robot presenters. Thus, the experiment results partially supported research question 3; the human presenter’s touching behavior enhanced the presenter’s *kawaii* feelings toward the object and the observer’s *kawaii* feelings toward the object without enhancing the observer’s *kawaii* feelings toward the presenter (Fig 13). The results in the robot presenter condition again partially supported research question 1. In other words, Experiment II’s results replicated the results of Experiment I; although the touch behaviors effectively increased the feelings of *kawaii* regardless of the presenter types, no *kawaii-*triangle phenomenon was observed in this setting.

### Why did the participants experience lower *kawaii* feelings for the doll with a human presenter?

In this experiment, when the presenter was a human, we believe the questionnaire items related to the feelings of *kawaii* toward the doll were lower for the following two main reasons: limited modality as a negative impact for the human presenter and the appearance of the presented item as a positive impact for the robot presenter. For the former, the human presenter in Experiment II wore a mask to hide her facial expressions because our robot presenter did not have such an ability. So we needed to avoid the effects of facial expressions, although a past study showed the importance of smiling to express *kawaii* feelings [8]. The lack of facial expressions decreased the perceived *kawaii* feelings of the human presenter. Concerning the latter, the item’s appearance is based on the robot presenter, unlike the human presenter. A situation in which a robot introduces a doll that looks like itself may have been perceived as more comical and favorable than a situation in which a human presenter introduced a certain doll. Note that although the situation is limited, this result shows one advantage of a robot presenter over a human presenter in informational tasks.

## General discussion

### Different modalities and relationships

In this study, we investigated the influences of viewing a touch behavior toward feelings of *kawaii*. Using non-static visual stimuli is one unique point compared to past related studies that used static visual stimuli (e.g., pictures) [8, 23, 24]; in this context, using different modalities increases such feelings through non-static visual stimuli. For example, as described above, a past study showed the importance of smiling to express feelings of *kawaii* [8]. Recent robotics researchers developed robots with rich DOFs for facial expressions [31–36]. Therefore, their smiling behaviors can be used during information-providing tasks.

Moreover, one possible future work is investigating the effects of smiling behaviors toward the *kawaii*-triangle phenomenon. Our study successfully expressed the *kawaii* feeling of presenters using touch behaviors, but that feeling did not increase toward presenters. If adding smiling behaviors with the same experimental setting as our experiment increases the *kawaii* feeling both toward presenters and the object, smiling behaviors might be an essential factor to elicit the *kawaii*-triangle phenomenon. Another useful modality is their speeches. For example, related works described the importance of pitch during gestures to emphasize the salience of information [25, 26]. These research works did not focus on feelings of *kawaii*, although such speech characteristics could have influenced them.

Another interesting point is investigating the perceived *kawaii* feelings of others in third-party relationships among a target, an observer, and a presenter. Past studies mainly investigated the *kawaii* feeling of an observer toward a target, i.e., only their one-to-one relationship. On the other hand, our study investigated the perceived *kawaii* feelings of presenters toward a target. Our results showed evidence that social robots are capable of conveying *kawaii* emotions to people, similar to a human presenter. The number of people and robots in the groups might provide different social effects, such as peer pressure [37–39]. Their personal relationships might also influence their perceived *kawaii* feelings.

### Limitations

This study suffers from several limitations, including just using a specific robot (i.e., Pepper), dealing with a specific feeling (i.e., *kawaii*), and heuristic parameters for behavior designs. Our possible future works will investigate touch behavior effects with different robots, feelings, and parameters. Moreover, the robot’s hardware limitations complicated designing strong aggression behaviors to resemble those of humans. Related to the cute aggression effects, we only investigated the perceived *kawaii* feelings without emphasized behaviors in Experiment II due to the pros and cons of such a behavior design. However, regardless of these limitations, our study provides value for robotics researchers who are interested in touch behavior effects and the designs of information-providing tasks. In fact, Pepper is already a common social robot platform for robotic-shopping assistants [14]. Therefore, the knowledge from our study can be easily applied to them. Our study can also provide scaffolding for future research that probes *kawaii* behavior designs. If researchers want to implement behaviors for *kawaii* expressions, our knowledge will help them prepare a baseline.

We also experimented with a specific doll as a target object. The basic *kawaii* feeling of a target object might influence the effects of presentation styles. In fact, the doll’s average *kawaii* feeling is only less than neutral in the no-touch and normal conditions in Experiment I. However, the values in the other conditions exceed neutral. We believe this phenomenon suggests that the perceived *kawaii* feeling for the object is influenced by other people’s behaviors toward the object, such as presentation, as we tested in the experiment. We did not investigate the basic *kawaii* feeling for the doll without behaviors of the robot or the human presenter in this experiment. Therefore, it remains unknown whether the no-touch and normal conditions increased the perceived *kawaii* feeling more than just showing the doll. Moreover, if the basic *kawaii* feeling for an object is quite low, the behaviors of the others might not be effective.

Since we only conducted our experiment with Japanese participants, generality and cultural differences should be considered. Even though *kawaii* is an example of a typical element of Japanese culture and such concepts have been spreading worldwide, past studies reported cultural differences about *kawaii* feelings between China and Japan [40] and Israeli and USA [41, 42]. These studies compared the perceived differences of *kawaii* to Japan, although they also reported that these other cultures held positive views of *kawaii*/cute concepts. Therefore, we believe this research will contribute to building a basis of comparison for the perception of *kawaii* in human-robot and human-human interaction research topics in the context of investigating cultural differences.

Another limitation is that we only focused on a user study, i.e., no discussion of a detailed methodology that naturally enables robots to touch items. We believe that our results will be one piece of evidence that explains why social robots need to touch items. But the implementation parts have not been covered yet. However, since many researchers are already manipulating research topics with robot arms [43, 44], using their knowledge will be useful to achieve autonomous social robots that naturally touch items. Related to this point, since we only distributed evaluation questionnaires in this study, using additional quantitative measures is important. For example, analyzing facial expressions, gaze behaviors, and physiological measurements might provide interesting implications.

## Conclusion and future directions

We described how the influence of viewing a presenters’ touching behavior toward an object and its motion styles increases the feeling of *kawaii* and improves the impressions of the presentation itself in the context of an information-providing task. We conducted two kinds of experiments, and both of our results showed that viewing touching behaviors increased feelings of *kawaii* and wanting to approach the doll compared to non-touching behaviors. The presenter’s impression of the doll also increased more than the normal style of non-touching without any significant effects on the presenters. These results provide evidence for using touch behaviors to express feelings of *kawaii* and increase such perceived feelings toward specific items for both human and robot presenters.

Existing studies on *kawaii* feelings have mainly focused on static images, locomotion behaviors, and one-to-one relationships between target objects and observers. But in this study, we investigated the effects of touching behaviors and third-party relationships among a target, an observer, and a presenter. Our proposed framework will provide new insight to understand *kawaii* feelings, which can be useful for investigating factors and relationships that are not dealt with in this study, such as the appearances of presenters, generality, and cultural difference. It also offers a basis for future methodological directions of *kawaii* research studies.

## Supporting information

S1 File Anonymized data set. (XLSX)

S2 File (PDF)
