# Supplementary material for: Kawaii emotions in presentations: Viewing a physical touch affects perception of affiliative feelings of others toward an object
Source: PLoS One. 2022 Mar 24;17(3):e0264736. doi: 10.1371/journal.pone.0264736 (PMC8946741; doi:10.1371/journal.pone.0264736)
Supplement: S2 File — (PDF) [file pone.0264736.s002.pdf]

2020 IEEE/RSJ International Conference on Intelligent Robots and Systems (IROS)
October 25-29, 2020, Las Vegas, NV, USA (Virtual)

Can a Robot's Touches Express the Feeling of Kawaii toward an Object?

Yuka Okada, Mitsuhiko Kimoto, Takamasa Iio, Katsunori Shimohara, Hiroshi Nittono, and Masahiro Shiomi

***Abstract*—*Kawaii*, a Japanese word that means "cute," is an essential design concept in consumer and pop culture in Japan. In this study, we focused on a situation where a social robot describes an object during an information-providing task, which is commonly required for social robots in daily environments. Since past studies reported *kawaii* feelings are associated with a motivation to approach a target, our robot expressed feelings of *kawaii* to objects touch behaviors. We also focused on whether touch behaviors that emphasize style increase the feeling of *kawaii* of the touched object, following a phenomenon where people strongly touch a target when they overwhelmingly feel positive emotion: *cute aggression*. Our experimental results showed the effectiveness of touch behaviors to express the feelings of *kawaii* from the robot toward objects and to increase the participants' feelings of *kawaii* toward the object. We identified fewer effects from the participants to the robot. The emphasized motion style did not show any significant effects for the *kawaii* feelings.**

I. Introduction

Several human science works report that people treat cute things favorably, and cute stimuli provide positive feelings, change behaviors, and encourage interaction [1-5]. In fact, we can find many scenes where cuteness encourages interaction between people, e.g., a baby's casual behaviors evoke parental smiles, a grandchild's gentle words cheer up grandparents, and walking a dog attracts people and promotes conversation.

For social robots that work in daily environments, the concept of cuteness is essential [6][7]. We believe that there are two approaches to deal with cuteness for social robots: 1) increasing the intrinsic cuteness of the robot's appearance and motion designs and 2) communicating the cuteness of others by robot's behaviors. The former idea is already employed in the context of consumer purposes, i.e., where recent social robots exploit cute designs for their appearances and behaviors. Particularly in Japan, companies are focusing on *kawaii* (a Japanese word that means "cute" that has positive connotations [5, 8]) concepts when designing both robot appearances and behaviors. Examples of such robots include Paro, LOVOT, and Robohon. The *kawaii* concept is a critical factor in Japanese commercial aspects and pop culture [9][10].

[1]This research work was supported in part by JST CREST Grant Number JPMJCR18A1, Japan JSPS KAKENHI Grant Numbers 18H03311, and JP 19J01290, and JST, PRESTO Grant Number JPMJPR1851, Japan

Y. Okada, M. Kimoto, T. Iio, K.Shimohara, H. Nittono and M. Shiomi are with ATR, Kyoto, Japan. (e-mail: m-shiomi@atr.jp).
Y. Okada and K. Shimohara are also with Doshisha Univ., Kyoto, Japan. (e-mail: okada2019@sil.doshisha.ac.jp)
M. Kimoto is also with Keio Univ., Tokyo, Japan.
T. Iio is also with University of Tsukuba / JST PRESTO, Ibaraki, Japan.
H. Nittono is also with Osaka Univ., Osaka, Japan.

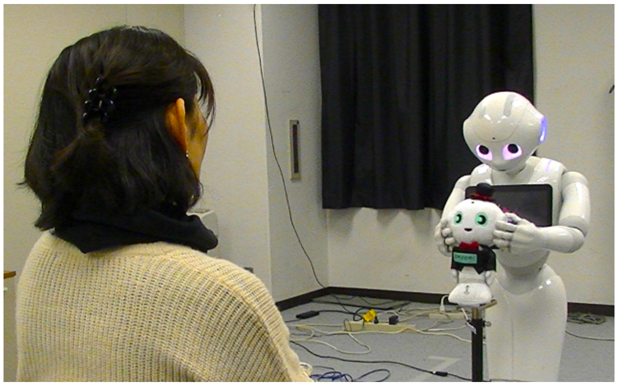

Figure 1. Pepper describes a doll by touching it for emphasis

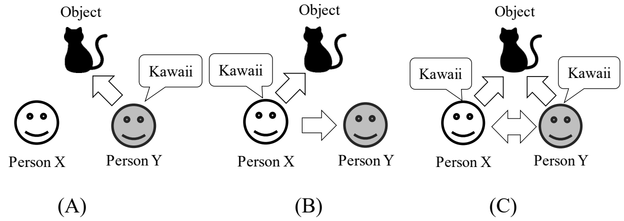

Figure 2. *Kawaii* triangle concept [8]

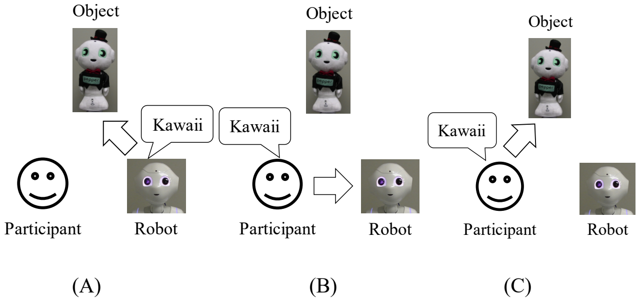

Figure 3. Our hypotheses

In this study, we focus on an approach that conveys the cuteness of others through a robot's behaviors. We believe that conveying cuteness or the feeling of *kawaii* to others has value in the context of information-providing tasks, which are commonly done by social robots that work in daily environments [11-13]. For example, when a robot works as a clerk at a shopping mall, it needs to recommend items by describing them to customers, such as a food's taste, the usefulness of a gadget, an object's economical cost, a doll's cuteness, etc. Therefore, we focused on a situation where a robot expresses an object's *kawaii* toward an observer (Fig. 1).

How does a robot emphasize the *kawaii* of objects? Related research of *kawaii* identified an interesting concept of social effects called the "*kawaii* triangle." [8] (Fig. 2). When

person X observes the smiles of person Y based on the *kawaii* aspects (Fig. 2-A) of an object (e.g., a cat), person X may have positive impressions about person Y. Then person X will also have similar feelings about person Y, e.g., smiling together. Such interaction might increase person X's feeling of *kawaii* toward the object (Fig. 2-B). Moreover, expressing this feeling might further enhance person Y's impression of person X and the object, vice versa (Fig.2-C). Following this concept, we hypothesized that if the robot expresses a greater feeling of *kawaii* toward an object (Fig.3-A), an observer will perceive more *kawaii* for both the robot (Fig.3-B) and the object (Fig.3-C). Note that we did not focus on the feeling of *kawaii* from the robot to the participant in this study.

To express *kawaii* to an object by a robot, we employed touch behaviors during the robot's explanations. Because the feeling of *kawaii* is associated with a motivation to approach a target, touch behaviors create a situation where the robot's body part (i.e., a hand) closely approaches the target. Other studies identified a phenomenon called *cute aggression* that captures the relationship between touch and feelings of *kawaii* [14, 15]. These studies reported behaviors or attitudes toward cute things, e.g., participants felt like "when I look at this baby, I feel like pinching her cheeks or being playfully aggressive" [15]. Thus, we also hypothesized that if a robot exaggeratedly touches an object to express its feeling of *kawaii*, a participant who observes the object and the robot might perceive a greater feeling of *kawaii* to the object and the robot.

We investigate whether a robot's touch behaviors and its exaggeration to an object increase the perceived feeling of *kawaii* of the robot toward the object as well as such feelings of the participants toward the object and the robot. We prepared two explanations behaviors (*touch* and *no-touch*) and two motion types (*normal* and *emphasized*) with a Pepper robot and a Pepper doll as an object and addressed the following two research questions:

- Can a robot's touch behaviors increase the perception of a robot's feeling of *kawaii* toward an object and an observer's feeling of *kawaii* toward the robot and the object?

- Are exaggerated of explanatory behaviors perceived as a stronger feeling of *kawaii* of the robot toward the object? Do they induce an observer's feeling of *kawaii* toward the object and the robot?

II. Related works

To naturally and understandably provide information, both the gestures and the speech contents of the presenters are essential. Robotics researchers developed several functions for conversational interaction by analyzing human-human interactions to enable social robots to deal with human-like information-providing tasks: gaze behaviors [16, 17], body gestures [18-21], spatial coordination [22, 23], approaching people [24], and distribution [25, 26].

In the context of the emphasis effects of gestures, Bremner et al.'s investigation is pioneering into the integration effects of the robot's gestures and speeches [27, 28]. They reported that a robot's beat gestures less effectively convey salience information than a human based on speech patterns through pitch emphasis. They also reported that people understand iconic gestures. Other studies investigated the effectiveness of deictic gestures to emphasize salience information, e.g., pointing to text blocks and underlining a word or phrase in a medical context. These functions were implemented for computer-based graphics agents to empower hospital patients who have low health literacy [29, 30]. These studies provided rich knowledge to achieve more natural and effective behavior designs for social robots that provide information to people.

However, these studies focused less on a situation where social robots explain objects by touching them. In other words, the effects of touch behaviors remain unknown in the context of information providing contexts. Even though human-robot touch interaction is a growing research topic, researchers are mainly focusing on its positive effects on physical/mental supports, such as therapy purposes [31-35]. Other studies focused on expressing basic emotions and intimacy by touching from interacting persons [36, 37] without investigating the effects of touch behaviors in information-providing contexts and expressing a feeling of *kawaii*.

Touch behaviors are related to expressing the feeling of *kawaii* because such feelings are associated with a motivation to approach a target and are related to aggressive touch behaviors [5, 8, 14]. Past studies reported the positive effects of *kawaii* on people's behavioral changes [4, 5], providing positive feelings [1], and attractiveness [2, 3]. If social robots can emphasize the feeling of *kawaii* toward objects to people, that result would be useful for information-providing tasks.

Note that the baby scheme is one famous design policy for expressing *kawaii* [38, 39]. Such a design policy has been adapted to several commercial products, including such social robots as Paro, LOVOT, and Robohon. But these design policies have mainly focused on the appearance of objects, not on how to express the feeling of *kawaii* to others. Thus, our study has the following two unique points compared to past related studies: 1) it investigated the relationship between touch behaviors and the feeling of *kawaii*, and 2) it investigated the relationship between the exaggeration of the robot's motions, including touching, and feelings of *kawaii*.

III. Robot for Experiment

A. Robot and its information-providing task

In this study, we used Pepper (Softbank Robotics, Fig. 4-A) to describe an object. The robot has 20 degrees of freedoms (DOFs): two DOFs for its head, six in each arm, and six for its lower body. It is 121 cm high.

We used a doll that resembles Pepper as an object (Fig. 4-B). We put it in front of the robot, and a participant sat in front of the doll (Fig. 4-C), which was 28 cm high. We placed it on a 65-cm high stand to adjust its height. In the information-providing task, the robot first introduces itself to the participants and then explains the doll's four characteristics: its costume, its sense of touch, its shape, and its face design. Table I shows the speech contents for each part.

B. Touch/no-touch behaviors

Since we are investigating the effects of touch behaviors to express the feeling of *kawaii*, we prepared two behaviors: *touch* and *no-touch*.

1) Touch behavior

We prepared four different touch behaviors to explain four different parts of the doll: costume, touch feeling, shape, and face design (Table II). For the costume behavior, the robot touches the doll's body with both hands. For the sense of touch, it strokes the doll's head with its right hand. For the shape behavior, it touches the doll's foot with its left hand. For the face design, the robot squeezes the doll's cheeks with both hands.

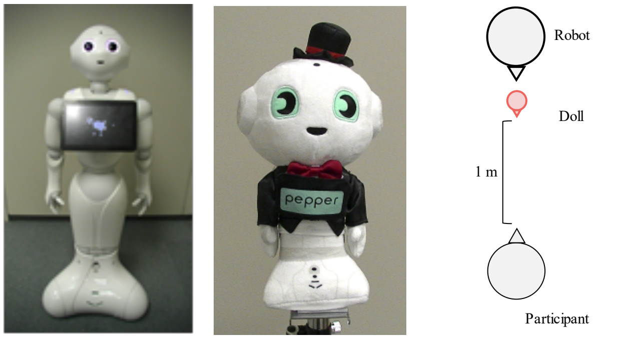

(A) Pepper (B) Doll (C) Experiment setting

Figure 4. Robot, doll, and information-providing setting

TABLE I. SPEECH CONTENTS FOR EACH PART OF DOLL

Part	Speech contents
Costume	Unlike me, this Pepper wears a tuxedo. Very stylish.
The sense of touch	It feels fluffier and cuddlier than me.
Shape	Although I don't really think I'm fat, this doll's shape does resemble me.
Face design	I like its big face and round eyes.

TABLE II. TOUCH/NO-TOUCH BEHAVIORS CONTENTS

Part	Robot behaviors	
	Touch	***No-touch***
Costume	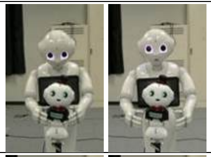	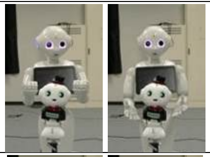
The sense of touch	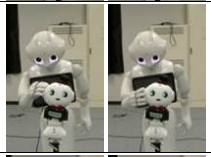	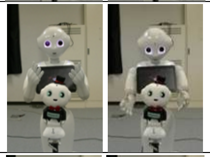
Shape	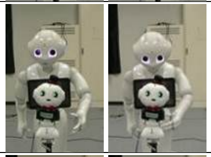	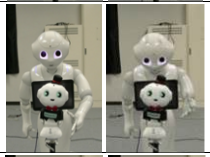
Face design	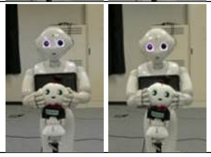	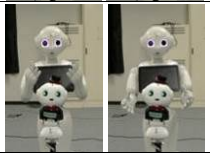

2) No-touch behavior

We also prepared four different no-touch behaviors, including spreading its hands around the target part and/or moving them to emphasize the doll's shape (Table II), because past studies reported that deictic and iconic gestures (instead of beat gestures) are useful for information providing [27-30]. Note that the robot is spreading its hands to attract attention instead of a pointing gesture (i.e., deictic gesture) due to the hardware limitations of its hands.

The robot spreads both hands around the doll's body for the costume and the sense of touch behaviors. It alsospreads both hands around the doll's body and emphasizes its body or face shape to explain the shape and face design behaviors.

C. Motion style

We also investigated the effects of motion styles in the context of expressing a feeling of *kawaii*, based on the concept of *cute aggression* [14]. We prepared two motion styles: *normal* and *emphasized*.

1) Normal

In this condition, we determined the speed of the robot's body movements based on observations of the people's explanatory behaviors. We conducted a preliminary data collection where three participants described the doll and recorded their body gestures. We heuristically adjusted the speeds of the robot's body movements by imitating the observed participant gestures as closely as possible.

2) Emphasized

In this condition, we increased the speed of the movements of the robot's body parts to emphasize its descriptions. We also heuristically adjusted the speed ratio of the robot gestures and ultimately chose a speed that was three times as fast as the *normal* condition.

In this study, we only focused on the expressions of gestures, i.e., not on speech characteristics even though a past study stressed the importance of pitch emphasis for this purpose [29, 30]. Our study is investigating the effects of the robot's touch behaviors and its motion style to express a feeling of *kawaii* by considering the *cute aggression* concept, not the integration effects of gestures and speech.

IV. EXPERIMENT

A. Hypothesis and prediction

The expressions of *kawaii* previously mediated the *kawaii* feelings of others [5, 8]. Feelings of *kawaii* are also associated with the motivation to approach an object. Perhaps a robot's touch behaviors express its *kawaii* feeling toward the object because its body part is close to the target during the touch behavior.

Perceiving the robot's feeling of *kawaii* toward an object will mediate that feeling of the participants toward both the object and the robot. The feeling of *kawaii* will lead to more positive evaluations about the robot as well as its descriptions. Increasing the feeling of *kawaii* will also raise the motivation of the observer to approach the object. Based on these considerations, we made the following four predictions about these effects:

Prediction 1: The robot's touch behaviors will increase the perception of the robot's feelings of *kawaii* and the motivation to approach the object (Fig.3-A) compared to the no-touch behaviors.

Prediction 2: The robot's touch behaviors will increase the participants' feelings of *kawaii* and their motivation to approach the robot (Fig.3-B) compared to the no-touch behaviors.

Prediction 3: The robot's touch behaviors will increase the participants' feelings of *kawaii* and their motivation to approach the object (Fig.3-C) compared to the no-touch behaviors.

Prediction 4: Participants positively evaluate the robot with touch behaviors compared to the robot without touch behaviors.

In addition, we are interested in a concept related to the feeling of *kawaii* and touch behaviors: *cute aggression* [14]. Past studies reported that people strongly touch an object when they feel overwhelmingly positive emotion. If the robot touches the doll with an emphasized style, will the feeling of *kawaii* increase? To answer this question, we also made another prediction:

Prediction 5: The robot's touch behaviors with an emphasized style will increase the perceived feeling of *kawaii* and motivation to approach the object more than with the normal style.

B. Conditions

This study had within-participant designs. All participants experienced four trials: combinations of two touch factors (*touch* and *no-touch*) and two motion factors (*normal* and *emphasized*). The order was counterbalanced.

1) Touch factor

***Touch* condition**: the robot used touch behaviors during its descriptions (Section III. B-1).

***No-touch* condition**: the robot used no-touch behaviors during its descriptions (Section III. B-2).

2) Motion factor

***Normal* condition**: the speed of the robot's body movements was based on observations of human behaviors (Section III. C-1).

***Emphasized* condition**: the robot used a speed that is three times faster than the *normal* condition (Section III-C-2).

C. Procedures

At the beginning of the experiment, the participants gave written informed consent to join this study, which was approved by the ethics committee of our institute. Then the experimenter clearly explained its procedures and told the participants to imagine the following situation: a robot as a clerk is recommending a doll to you as a customer.

In all the conditions, the robot introduced itself, described each part of the doll using different behaviors based on the conditions, and finally concluded the interaction. The participants filled out questionnaires after each trial.

D. Participants

Forty-two participants (21 females and 21 males, ranging in age from 21 to 49, the average age was 37.83, S.D. was 7.92) joined our experiment.

F. Measurements

To investigate the effects of the touch and motion factors, we measured by questionnaires two subjective items related to *kawaii* feelings and four subjective items related to their perceived impressions of the robot and its descriptions.

For the first two items, we employed two existing items: the degree of *kawaii* and the degree of *wanting to approach* [5][8]. Past studies reported that a feeling of *kawaii* is associated with a motivation to approach a target. Both items were measured by three topics: the perceived robot's feelings toward the doll, the participant's feelings toward the doll, and the participant's feelings toward the robot. Thus, we measured six items about their feelings of *kawaii*.

For the latter four items, we employed one existing scale (*likeability* of five items [40]), one item about the degree of a *good description*, and two items about the naturalness of the whole motions, and the hand motions individually. All items were evaluated on a 1-to-7-point scale, where 1 is the most negative and 7 is the most positive.

V. Results

A. Analysis of questionnaire results

Figure 5-A shows the questionnaire results about the perceived robot's feeling of *kawaii* toward the doll. We conducted a two-way repeated measures ANOVA whose results showed a significant difference in the touch factor ($F(1, 41) = 19.658$, $p < 0.001$, *partial* $\eta^2 = 0.324$). We did not find any significant differences in the motion factor ($F(1, 41) = 1.474$, $p = 0.232$, *partial* $\eta^2 = 0.035$) or in their interaction ($F(1, 41) = 2.269$, $p = 0.140$, *partial* $\eta^2 = 0.052$).

Figure 5-B shows the questionnaire results about the perceived robot's *wanting to approach* toward the doll. We conducted a two-way repeated measures ANOVA whose results showed a significant difference in the touch factor ($F(1, 41) = 13.847$, $p = 0.001$, *partial* $\eta^2 = 0.252$) and a significant trend in the motion factor ($F(1, 41) = 2.921$, $p = 0.095$, *partial* $\eta^2 = 0.067$). There was no significant difference in their interaction ($F(1, 41) = 0.171$, $p = 0.681$, *partial* $\eta^2 = 0.004$).

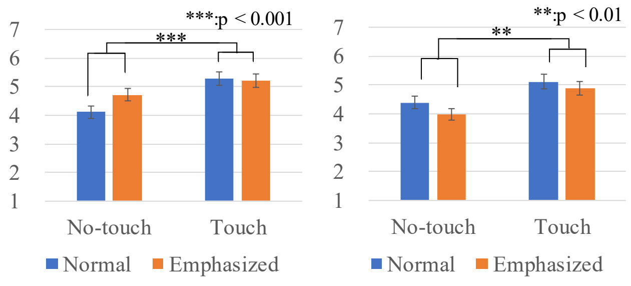

(A) *kawaii* (B) *wanting to approach*

Figure 5. Questionnaire results of perceived robot's feeling of *kawaii* and *wanting to approach* doll (average and S.E.)

Figure 6-A shows the questionnaire results about the *kawaii* feeling toward the robot. We conducted a two-way repeated measures ANOVA whose results did not show significant differences in the touch factor ($F(1, 41) = 2.821$, $p = 0.101$, *partial* $\eta^2 = 0.064$), in the motion factor ($F(1, 41) = 0.198$, $p = 0.658$, *partial* $\eta^2 = 0.005$), or in their interaction ($F(1, 41) = 0.125$, $p = 0.725$, *partial* $\eta^2 = 0.003$).

Figure 6-B shows the questionnaire results about the *wanting to approach* toward the robot. We conducted a two-way repeated measures ANOVA whose results showed a significant trend in the touch factor ($F(1, 41) = 3.297$, $p = 0.077$, *partial* $\eta^2 = 0.074$). We did not find significant differences in the motion factor ($F(1, 41) = 1.911$, $p = 0.174$, *partial* $\eta^2 = 0.045$) and in their interaction ($F(1, 41) = 0.090$, $p = 0.766$, *partial* $\eta^2 = 0.002$).

Figure 7-A shows the questionnaire results about the participant's feeling of *kawaii* toward the doll. We conducted a two-way repeated measures ANOVA whose results showed significant differences in the touch factor ($F(1, 41) = 24.752$, $p < 0.001$, *partial* $\eta^2 = 0.376$), in the motion factor ($F(1, 41) = 13.478$, $p = 0.001$, *partial* $\eta^2 = 0.247$), and in their interaction ($F(1, 41) = 9.696$, $p = 0.003$, *partial* $\eta^2 = 0.191$). Multiple comparisons with the Bonferroni method showed a significant difference in the *normal* condition (*touch* > *no-touch*, $p < 0.001$) and the *no-touch* condition (*emphasized* > *normal*, $p < 0.001$).

Figure 7-B shows the questionnaire results about the participant's feeling of *wanting to approach* toward the doll. We conducted a two-way repeated measures ANOVA whose results showed significant differences in the touch factor ($F(1, 41) = 36.896$, $p < 0.001$, *partial* $\eta^2 = 0.474$), in the motion factor ($F(1, 41) = 17.104$, $p < 0.001$, *partial* $\eta^2 = 0.294$), and in their interaction ($F(1, 41) = 12.454$, $p = 0.001$, *partial* $\eta^2 = 0.233$). Multiple comparisons with the Bonferroni method showed a significant difference in the *normal* condition (*touch* > *no-touch*, $p < 0.001$) and in the *no-touch* condition (*emphasized* > *normal*, $p < 0.001$).

Figure 8-A shows the questionnaire results about participants' *likeability* toward the robot. We conducted a two-way repeated measures ANOVA whose results showed a significant difference in the touch factor ($F(1, 41) = 7.441$, $p = 0.009$, *partial* $\eta^2 = 0.154$). We did not find significant differences in the motion factor ($F(1, 41) = 0.004$, $p = 0.950$, *partial* $\eta^2 = 0.000$) or in their interaction ($F(1, 41) = 0.018$, $p = 0.895$, *partial* $\eta^2 < 0.001$).

Figure 8-B shows the questionnaire results about the participants' feelings about the descriptions of the robot's presentation. We conducted a two-way repeated measure ANOVA whose results showed a significant difference in the touch factor ($F(1, 41) = 7.192$, $p = 0.011$, *partial* $\eta^2 = 0.149$). We did not find significant differences in the motion factor ($F(1, 41) = 0.406$, $p = 0.528$, *partial* $\eta^2 = 0.010$) or in their interaction ($F(1, 41) = 0.803$, $p = 0.376$, *partial* $\eta^2 = 0.019$).

Figure 9-A shows the questionnaire results about the naturalness toward the entirety of the robot's motions. We conducted a two-way repeated measures ANOVA whose results showed significant differences in the motion factor ($F(1, 41) = 9.257$, $p = 0.004$, *partial* $\eta^2 = 0.184$) and in the touch factor ($F(1, 41) = 4.424$, $p = 0.042$, *partial* $\eta^2 = 0.097$). We did not find significant differences in their interaction ($F(1, 41) = 0.134$, $p = 0.716$, *partial* $\eta^2 = 0.003$).

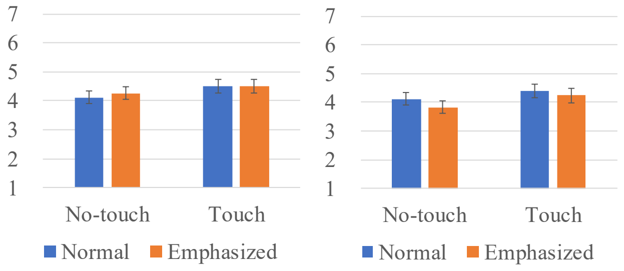

(A) *kawaii* (B) *wanting to approach*

Figure 6. Questionnaire results of participant's feeling of *kawaii* and *wanting to approach* robot (average and S.E.)

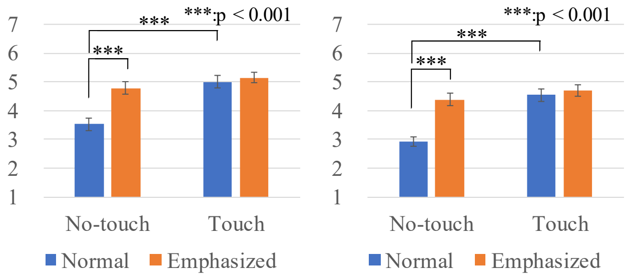

(A) *kawaii* (B) *wanting to approach*

Figure 7. Questionnaire results of participant's feeling of *kawaii* and *wanting to approach* doll (average and S.E.)

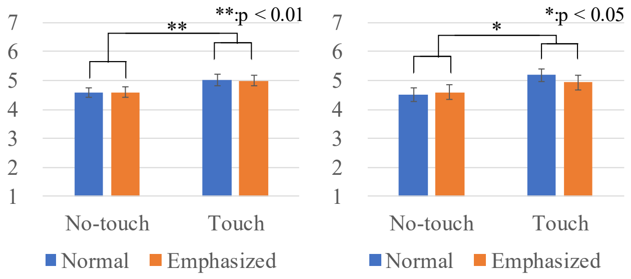

(A) *Likeability* (B) *Presentation*

Figure 8. Questionnaire results of *likeability* and the feeling of a good explanation of robot's presentation (average and S.E.)

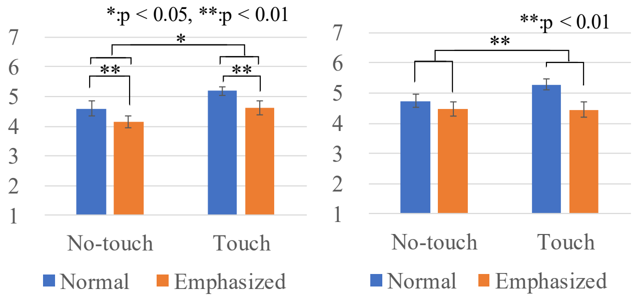

(A) *Whole motion* (B) *Hand motion*

Figure 9. Questionnaire results of *naturalness* of entirety of robot's motions and its hand motion during presentation (average and S.E.)

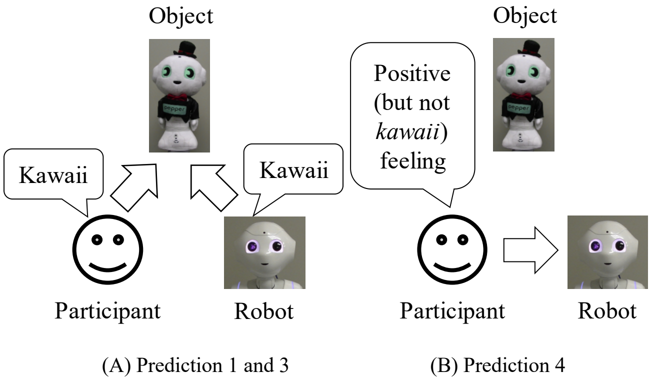

Figure 10. Illustration of supported predictions

Figure 9-B shows the questionnaire results about naturalness toward the robot's hand motions. For analysis, we conducted a two-way repeated measures ANOVA whose results showed a significant difference in the motion factor ($F(1, 41) = 9.670$, $p = 0.003$, *partial* $\eta^2 = 0.191$). We did not find significant differences in the touch factor ($F(1, 41) = 1.026$, $p = 0.317$, *partial* $\eta^2 = 0.024$) or in their interaction ($F(1, 41) = 2.144$, $p = 0.151$, *partial* $\eta^2 = 0.050$).

B. Summary of analysis

Our experiment results showed that a robot's touch behaviors increased the perception of its feeling of *kawaii* and the motivation to approach the doll compared to its no-touch behaviors. Its impressions of the robot to the doll also increased compared to its no-touch behaviors with the normal style. Our participants highly evaluated the robot's likeability and its explanations when it used the touch behaviors. On the other hand, the robot's touch behaviors did not increase the participants' feeling of *kawaii* or their motivation to approach the robot compared to the robot's no-touch behaviors with the normal style. Therefore, predictions 1, 3 (Fig. 10-A) and 4 (Fig. 10-B) are supported; prediction 2 is not supported.

On the other hand, our experiment results did not show any significant effect of the emphasized style in the touch behaviors; prediction 5 is not supported. Instead, the emphasized style is effective for the *no-touch* condition. Therefore, the robot's no-touch behaviors with an emphasized style increased the perceived feeling of *kawaii* and the motivation to approach the doll compared to the normal style as well as the participants' feelings. Note that the feelings of the naturalness of the robot's whole/hand motions were more highly evaluated in the *normal* condition compared to the *emphasized* condition, as well as in the *touch* condition compared to the *no-touch* condition.

VI. Discussion

A. Expressing kawaii feeling by social robots

Our experiment results suggest several implementations to express the feeling of *kawaii* for social robots. First, if a robot can touch an object during its descriptions or recommendations, such touch behaviors are beneficial because they effectively increase both the feelings of *kawaii* to the object and positive evaluations toward the robot's descriptions. Touch behaviors might be useful to express feelings of *kawaii* for virtual agents if their graphic systems can deal with collisions between such agents and objects.

On the other hand, unlike our assumption, our results did not show any advantages in the touch behaviors with emphasized style; they only decreased the naturalness of the robot's motions. Therefore, using the normal touch style is better. Note that our experimental settings for expressing *cute aggression* remain limited (Section VI. E), e.g., due to the robot's hardware limitation such as torque. Therefore, one future work will investigate *cute aggression* effects with different hardware. Evaluations with virtual agents might be useful to investigate *cute aggression* effects because their hardware settings are not limited.

Second, the results showed both the positive/negative perspectives of the emphasized style in the no-touch behaviors. Even if the emphasized style significantly decreased its naturalness, it increases several feelings of *kawaii*. These results suggest the effectiveness of the emphasized style in the no-touch behaviors based on the trade-off between the perceived naturalness and the feeling of *kawaii*.

From another perspective, investigating the touch effects with human experimenters is also a possible future work. If specific touch behaviors are effective for this purpose, data collection with human presenters and these implemented behaviors would contribute to a better understanding of the perception of *kawaii* feelings expressed by social robots.

B. Did the kawaii triangle occur?

The experiment results showed that participants had a greater feeling of *kawaii* toward the doll and perceived the robot's feeling of *kawaii* toward it when the robot used touch behaviors. However, their feelings of *kawaii* toward the robot did not increase. Thus, these results suggest that even if the robot's touch behavior enables it to increase its expression of a feeling of *kawaii* as well as the participants' feelings of *kawaii*, no *kawaii* triangle occurred in this study.

One possible reason is that the robot lacks modalities to express the feeling of *kawaii*. In this experiment, we focused on the differences between touch behaviors and the emphasized style of gestures. We used identical speech contents with identical characteristics. The robot could not change its facial expressions, e.g., smiling. A past study about the expressions of *kawaii* reported that smiling is one essential factor for expressing and perceiving a feeling of *kawaii* [8]. Therefore, the inability to express a smile (including laughter) might decrease this feeling to the robot. Another interesting future work is to investigate the effectiveness of a robot's smiling behaviors to express a deeper feeling of *kawaii*.

C. Possible modalities to express more kawaii feeling

As described above, we only focused on touch behaviors for expressing *kawaii*, but other possible modalities exist, of course. The advances of recent robotics hardware can achieve quite human-like appearances such as androids [41-43], and

using such robots' facial expressions would facilitate the expression of *kawaii* feelings. Even if their appearances are not human-like [44-46], their smile expressions might be useful.

Speech characteristics are also important to express a feeling of *kawaii*. In fact, past studies reported that integrating pitch characteristics with gestures effectively emphasizes salience information [27, 28]. Although such combinations do not focus on expressions of *kawaii*, they are one common approach of emphasis in information-providing contexts. They might also be useful for expressions of *kawaii*.

E. Limitations

Since we only used Pepper, generality about robot appearance and touch behaviors are limited. To apply our knowledge in actual environments, we need to investigate whether different kinds of robots can express a feeling of *kawaii* by their touch behaviors. Moreover, in this study, we heuristically and manually designed each touch behavior. Using the shape information of objects might autonomously enable robots to control their touch behaviors.

We only focused on the feeling of *kawaii* and its touch behaviors/styles based on past studies. Therefore, it is unknown whether touch behaviors are effective for expressing other feelings, such as cool, modern, and so on. Another future work will investigate the effects of touch behaviors during descriptions toward such different feelings.

However, even if several limitations exist, we believe that our study provides enough value for the human-robot interaction research field for investigating the effects of touch behaviors in an information-providing context.

VII. Conclusion

We focused on expressing the feeling of an object's *kawaii* by a robot's touch behaviors and its style in an information-providing context. Such a task is essential for social robots that work in daily environments, and *kawaii* is becoming an essential design concept in Japan. We experimentally investigated the perception of a robot's feeling of *kawaii* to an object as well as the participants' feeling of it to both the object and the robot by comparing touch/no-touch behaviors with a normal/emphasized style.

Our experiment results showed the advantages of touch behaviors compared to no-touch behaviors. The robot's touch behaviors increased the perceived robot's feeling of *kawaii* and their motivation to approach a doll as well as their impressions to it and likeability and positive impressions toward the robot's descriptions compared to no-touch behaviors with the normal style. The participants' feeling of *kawaii* toward the robot did not increase regardless of using touch behaviors. The emphasized motion style did not increase the feeling of *kawaii* for either the robot or the participants when the robot uses touch behaviors. It decreased the naturalness of the robot's motions but increased the feeling of *kawaii* if the robot used no-touch motions.

These experimental results provide useful knowledge about touch behavior design for social robots in the contexts of information providing and expressing the feeling of *kawaii*. Moreover, the results showed both positive and negative perspectives of the emphasized motion style for no-touch behaviors in the context of information providing, even if such style did not have a positive perspective for touch behaviors in this study.

Acknowledgment

We thank Sayuri Yamauchi for her help during the execution of our experiments.

References

[1] J. G. Myrick, "Emotion regulation, procrastination, and watching cat videos online: Who watches Internet cats, why, and to what effect?," Computers in human behavior, vol. 52, pp. 168-176, 2015.

[2] A. M. Proverbio, V. D. Gabriele, M. Manfredi, and R. Adorni, "No race effect (ORE) in the automatic orienting toward baby faces: When ethnic group does not matter," Psychology, vol. 2, no. 09, pp. 931-935, 2011.

[3] H. Nittono, and N. Ihara, "Psychophysiological responses to kawaii pictures with or without baby schema," SAGE Open, vol. 7, no. 2, pp. 2158244017709321, 2017.

[4] K. Nishiyama, K. Oishi, and A. Saito, "Passersby Attracted by Infants and Mothers' Acceptance of Their Approaches: A Proximate Factor for Human Cooperative Breeding," Evolutionary Psychology, vol. 13, no. 2, pp. 147470491501300210, 2015.

[5] H. Nittono, M. Fukushima, A. Yano, and H. Moriya, "The power of kawaii: Viewing cute images promotes a careful behavior and narrows attentional focus," PloS one, vol. 7, no. 9, pp. e46362, 2012.

[6] C. Caudwell, C. Lacey, and E. B. Sandoval, "The (Ir) relevance of Robot Cuteness: An Exploratory Study of Emotionally Durable Robot Design," in Proceedings of the 31st Australian Conference on Human-Computer-Interaction, pp. 64-72, 2019.

[7] C. Caudwell, and C. Lacey, "What do home robots want? The ambivalent power of cuteness in robotic relationships," Convergence, pp. 1354856519837792, 2019.

[8] H. Nittono, "The two-layer model of'kawaii': A behavioural science framework for understanding kawaii and cuteness," East Asian Journal of Popular Culture, vol. 2, no. 1, pp. 79-96, 2016.

[9] S. Kinsella, "Cuties in japan," Women, media and consumption in Japan, pp. 230-264: Routledge, 2013.

[10] S. Lieber-Milo, and H. Nittono, "From a Word to a Commercial Power: A Brief Introduction to the Kawaii Aesthetic in Contemporary Japan," Innovative Research in Japanese Studies, vol. 3, pp. 13-32, 2019.

[11] M. Niemelä, P. Heikkilä, H. Lammi, and V. Oksman, "A social robot in a shopping mall: studies on acceptance and stakeholder expectations," Social Robots: Technological, Societal and Ethical Aspects of Human-Robot Interaction, pp. 119-144: Springer, 2019.

[12] T. Iio, S. Satake, T. Kanda, K. Hayashi, F. Ferreri, and N. Hagita, "Human-Like Guide Robot that Proactively Explains Exhibits," International Journal of Social Robotics, 2019.

[13] P. Heikkilä, H. Lammi, M. Niemelä, K. Belhassein, G. Sarthou, A. Tammela, A. Clodic, and R. Alami, "Should a robot guide like a human? A qualitative four-phase study of a shopping mall robot," in International Conference on Social Robotics, pp. 548-557, 2019.

[14] O. R. Aragón, M. S. Clark, R. L. Dyer, and J. A. Bargh, "Dimorphous expressions of positive emotion: Displays of both care and aggression in response to cute stimuli," Psychological science, vol. 26, no. 3, pp. 259-273, 2015.

[15] K. K. Stavropoulos, and L. A. Alba, ""It's so cute I could crush it!": Understanding neural mechanisms of Cute Aggression," Journal of

Frontiers in behavioral neuroscience, vol. 12, pp. 300, 2018.
[16] B. Mutlu, T. Kanda, J. Forlizzi, J. Hodgins, and H. Ishiguro, “Conversational gaze mechanisms for humanlike robots,” ACM Transactions on Interactive Intelligent Systems (TiiS), vol. 1, no. 2, pp. 12, 2012.
[17] A. Yamazaki, K. Yamazaki, Y. Kuno, M. Burdelski, M. Kawashima, and H. Kuzuoka, “Precision timing in human-robot interaction: coordination of head movement and utterance,” in Proceeding of the twenty-sixth annual SIGCHI conference on Human factors in computing systems, Florence, Italy, pp. 131-140, 2008.
[18] B. Mutlu, J. Forlizzi, and J. Hodgins, “A storytelling robot: Modeling and evaluation of human-like gaze behavior,” in The 6th IEEE-RAS international conference on Humanoid robots, pp. 518-523, 2006.
[19] Y. Hato, S. Satake, T. Kanda, M. Imai, and N. Hagita, “Pointing to space: modeling of deictic interaction referring to regions,” in Human-Robot Interaction (HRI), 2010 5th ACM/IEEE International Conference on, pp. 301-308, 2010.
[20] T. Komatsubara, M. Shiomi, T. Kanda, and H. Ishiguro, “Can Using Pointing Gestures Encourage Children to Ask Questions?,” International Journal of Social Robotics, vol. 10, no. 4, pp. 387-399, 2017.
[21] A. Shimazu, C. Hieida, T. Nagai, T. Nakamura, Y. Takeda, T. Hara, O. Nakagawa, and T. Maeda, “Generation of Gestures During Presentation for Humanoid Robots,” in 2018 27th IEEE International Symposium on Robot and Human Interactive Communication (RO-MAN), pp. 961-968, 2018.
[22] F. Yamaoka, T. Kanda, H. Ishiguro, and N. Hagita, “A model of proximity control for information-presenting robots,” IEEE Transactions on Robotics, vol. 26, no. 1, pp. 187-195, 2010.
[23] C. Shi, M. Shiomi, T. Kanda, H. Ishiguro, and N. Hagita, “Measuring Communication Participation to Initiate Conversation in Human–Robot Interaction,” International Journal of Social Robotics, vol. 7, no. 5, pp. 889-910, 2015.
[24] S. Satake, T. Kanda, D. F. Glas, M. Imai, H. Ishiguro, and N. Hagita, “A robot that approaches pedestrians,” IEEE Transactions on Robotics, vol. 29, no. 2, pp. 508-524, 2013.
[25] M. Gharbi, P. V. Paubel, A. Clodic, O. Carreras, R. Alami, and J. M. Cellier, “Toward a better understanding of the communication cues involved in a human-robot object transfer,” in Robot and Human Interactive Communication (RO-MAN), 2015 24th IEEE International Symposium on, pp. 319-324, 2015.
[26] C. Shi, M. Shiomi, C. Smith, T. Kanda, and H. Ishiguro, “A Model of Distributional Handing Interaction for a Mobile Robot,” in Robotics: Science and Systems, pp., 2013.
[27] P. Bremner, and U. Leonards, “Speech and Gesture Emphasis Effects For Robotic and Human Communicators-a Direct Comparison,” in 2015 10th ACM/IEEE International Conference on Human-Robot Interaction (HRI), pp. 255-262, 2015.
[28] P. Bremner, and U. Leonards, “Iconic gestures for robot avatars, recognition and integration with speech,” Frontiers in psychology, vol. 7, pp. 183, 2016.
[29] T. Bickmore, L. Pfeifer, and L. Yin, “The role of gesture in document explanation by embodied conversational agents,” International Journal of Semantic Computing, vol. 2, no. 01, pp. 47-70, 2008.
[30] T. W. Bickmore, L. M. Pfeifer, and B. W. Jack, “Taking the time to care: empowering low health literacy hospital patients with virtual nurse agents,” in Proceedings of the SIGCHI conference on human factors in computing systems, pp. 1265-1274, 2009.
[31] R. Yu, E. Hui, J. Lee, D. Poon, A. Ng, K. Sit, K. Ip, F. Yeung, M. Wong, and T. Shibata, “Use of a Therapeutic, Socially Assistive Pet Robot (PARO) in Improving Mood and Stimulating Social Interaction and Communication for People With Dementia: Study Protocol for a Randomized Controlled Trial,” JMIR research protocols, vol. 4, no. 2, 2015.
[32] M. Shiomi, K. Nakagawa, K. Shinozawa, R. Matsumura, H. Ishiguro, and N. Hagita, “Does A Robot’s Touch Encourage Human Effort?,” International Journal of Social Robotics, vol. 9, pp. 5-15, 2016.
[33] H. Sumioka, A. Nakae, R. Kanai, and H. Ishiguro, “Huggable communication medium decreases cortisol levels,” Scientific Reports, vol. 3, pp. 3034, 2013.
[34] M. Shiomi, A. Nakata, M. Kanbara, and N. Hagita, “A Hug from a Robot Encourages Prosocial Behavior,” in Robot and Human Interactive Communication (RO-MAN), 2017 26th IEEE International Symposium on, pp. to appear, 2017.
[35] M. Shiomi, and N. Hagita, “Audio-Visual Stimuli Change not Only Robot’s Hug Impressions but Also Its Stress-Buffering Effects,” International Journal of Social Robotics, pp. 1-8, 2019.
[36] X. Zheng, M. Shiomi, T. Minato, and H. Ishiguro, “What Kinds of Robot's Touch Will Match Expressed Emotions?,” IEEE Robotics and Automation Letters, pp. 127-134, 2019.
[37] X. Zheng, M. Shiomi, T. Minato, and H. Ishiguro, “How Can Robot Make People Feel Intimacy Through Touch?,” Journal of Robotics and Mechatronics, vol. 32, no. 1, pp. (to appeear), 2019.
[38] K. Lorenz, “The innate forms of potential experience,” Z Tierpsychol, vol. 5, pp. 235-409, 1943.
[39] V. Brooks, and J. Hochberg, “A psychophysical study of" cuteness.",” Perceptual and Motor Skills, 1960.
[40] C. Bartneck, D. Kulić, E. Croft, and S. Zoghbi, “Measurement instruments for the anthropomorphism, animacy, likeability, perceived intelligence, and perceived safety of robots,” International Journal of Social Robotics, vol. 1, no. 1, pp. 71-81, 2009.
[41] T. Hashimoto, S. Hiramatsu, T. Tsuji, and H. Kobayashi, “Development of the face robot SAYA for rich facial expressions,” in 2006 SICE-ICASE International Joint Conference, pp. 5423-5428, 2006.
[42] S. Nishio, H. Ishiguro, and N. Hagita, “Geminoid: Teleoperated android of an existing person,” Humanoid robots: New developments, vol. 14, pp. 343-352, 2007.
[43] D. F. Glas, T. Minato, C. T. Ishi, T. Kawahara, and H. Ishiguro, “Erica: The erato intelligent conversational android,” in Robot and Human Interactive Communication (RO-MAN), 2016 25th IEEE International Symposium on, pp. 22-29, 2016.
[44] A. van Breemen, X. Yan, and B. Meerbeek, “iCat: an animated user-interface robot with personality,” in Proceedings of the fourth international joint conference on Autonomous agents and multiagent systems, pp. 143-144, 2005.
[45] M. Zecca, N. Endo, S. Momoki, K. Itoh, and A. Takanishi, “Design of the humanoid robot KOBIAN-preliminary analysis of facial and whole body emotion expression capabilities,” in Humanoids 2008-8th IEEE-RAS International Conference on Humanoid Robots, pp. 487-492, 2008.
[46] I. Lütkebohle, F. Hegel, S. Schulz, M. Hackel, B. Wrede, S. Wachsmuth, and G. Sagerer, “The bielefeld anthropomorphic robot head “Flobi”,” in 2010 IEEE International Conference on Robotics and Automation, pp. 3384-3391, 2010.
